# Effects of an imposed axial flow on a Ferrofluidic Taylor-Couette flow

**DOI:** 10.1038/s41598-019-51935-x

**Published:** 2019-10-28

**Authors:** Sebastian Altmeyer, Younghae Do

**Affiliations:** 1 Castelldefels School of Telecom and Aerospace Engineering, Universitat Politècnica de Catalunya, Barcelona, 08034 Spain; 2 Department of Mathematics, KNU-Center for Nonlinear Dynamics, Kyungpook National University, Daegu, 41566 Republic of Korea

**Keywords:** Applied mathematics, Fluid dynamics, Nonlinear phenomena

## Abstract

In this paper we investigate the effects of an externally imposed *axial mass flux* (*axial pressure gradient*, *axial through flow*) on ferrofluidic Taylor-Couette flow under the influence of either an axial or a transverse magnetic field. Without an imposed axial through flow, due to the symmetry-conserving axial field and the symmetry-breaking transverse field, it gives rise to various vortex flows in ferrofluidic Taylor-Couette flow such as wavy Taylor vortex flow (*wTVF*), wavy spiral vortex flow (*wSPI*) and wavy vortex flows (${\boldsymbol{wTV}}{{\boldsymbol{F}}}_{{{\boldsymbol{H}}}_{{\boldsymbol{x}}}}$ and ${\boldsymbol{wSP}}{{\boldsymbol{I}}}_{{{\boldsymbol{H}}}_{{\boldsymbol{x}}}}$), which are typically produced by a nonlinear interaction of *rotational*, *shear* and *magnetic* instabilities. In addition, when an axial through flow is imposed to a ferrofluidic Taylor-Couette flow in the presence of either an axial or a transverse magnetic field, *previously unknown new helical* vortex structures are observed. In particular, we uncover ‘*modulated Mixed-Cross-Spirals*’ with a combination of at least *three* different dominant azimuthal wavenumbers. Emergence of such new flow states indicates richer but potentially more controllable dynamics in ferrofluidic flows, *i*.*e*., an imposed axial through flow will be a new controllable factor/parameter in applications of a ferrofluidic and magnetic flows flow.

## Introduction

The understanding of the flow in the gap between concentric independently rotating cylinders (Taylor-Couette system, TCS) is both, of scientific interest, *i*.*e*., the understanding the various hydrodynamic stabilities^1,2^, and of practical interest for many engineering applications in rotating machinery. Specific examples to mention here include the lubricating flow between rotating shafts to be found in turbopumps in rocket engines, in multi-spool turbofan engines and in the bearing housing of low and high bypass aircraft engines^3,4^. Other areas of application are found in the bearing chambers of internal combustion aero-engines, rotating tube in heat exchangers, and the submerged pumps for water wells. Further motivation of this research are rotating filtration devices. In fact, rotating filtration, also used for blood filtration^5–7^, has been proposed for filtering suspensions and water purification via reverse osmosis^8–12^.

An axial mass flux (axial pressure gradient, axial through flow)^2,13–17^ typically allows to study fundamental and important problems in several engineering applications, e.g. journal bearings, biological separation devices, and rotating machinery. Through a systematic study of stabilities, bifurcations and spatiotemporal evolution of flow structures, the purpose of this paper is to demonstrate new flow states in a ferrofluidic TCS that have not been reported previously, and explore its potential use for flow control.

Recently there has been an increasing amount of interest in the flow dynamics of the TCS with a complex fluid^18–26^. Representative types of a complex fluids are ferrofluids^27^ for which recent studies unveiled the varieties and differences of ferrofluidic flows compared to classical fluids. Ferrofluids are manufactured fluids consisting of dispersion of magnetized nanoparticles in a liquid carrier. A ferrofluid can be stabilized against agglomeration through the addition of a surfactant monolayer onto the particles. In the absence of any magnetic field, the nanoparticles are randomly orientated so that the fluid has zero net magnetization. In this case, the nanoparticles alter little the viscosity and the density of the fluid. Thus, in the absence of any external field, a ferrofluid behaves as a simple (classical) fluid. However, when a magnetic field of sufficient strength is applied, the hydrodynamic properties of the fluid, such as the viscosity, can be *changed dramatically*^28,29^ and the *dynamics* can be drastically *altered*^18,20,26^. Ferrofluidic flows have wide applications, ranging from gaining insights into the fundamentals of geophysical flows through laboratory experiments^30,31^ to the development of microfluidic devices and computer hard drives. The additional option of applying a magnetic field provides further features for separation. For instance, using ferrofluids which are able to ‘stick’ on specific particles in fluid suspension, it is possible to separate such particles which do not differ in its specific weight.

**Figure 1:**
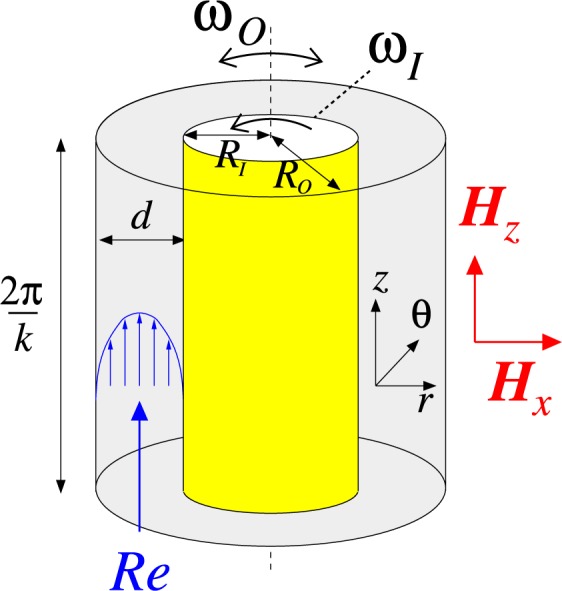
Schematics of TCS. Schematic sketch of the Taylor-Couette System in a homogeneous axial (transversal) magnetic field ${\bf{H}}={H}_{z}{{\bf{e}}}_{z}$ (${H}_{x}{{\bf{e}}}_{x}$) with an axial imposed mass flux *Re* (bottom to top). Note that the domain is periodic in the axial dimension in the present study.

A well established result of previous works is that under the influence of a symmetry-breaking transverse magnetic field, all flow states in the TCS become *intrinsically three-dimensional*^18,20,26^. In particular, a magnetic field has significant influence on the hydrodynamical stability and the underlying symmetries of the flow states through certain induced azimuthal modes^26^. It even induces and allows the study of turbulence at low Reynolds number^23^. In the present study, when an imposed axial through flow is presented in a ferrofluidic TCS (see Fig. 1), we discovered very complex flow states which are created by the interaction of three different instabilities; centrifugal instability due to rotation, shear instability due to axial mass flux and magnetic instability due to magnetic fields. That is, our main finding for such complex flow states is complex *localized* and toroidally closed wavy vortices (*wTVF*_*l*_, $wTV{F}_{l.{H}_{x}}$) as well as a new complex helical *modulated Mixed-Cross-Spirals* ($mMC{S}_{{H}_{x}}$). Interesting facts of the newly found modulated Mixed-Cross-Spirals are that (1) these states appearing between two wavy spirals are existed as *stable*, and (2) the number of stimulated azimuthal modes for these states is at least three, which are *first* described in this paper. As a result, the existence of multiple dominant azimuthal modes will create new types of helical states. We establish this striking result through extensive computations and bifurcation analyses of various flow states.

A common feature of a magnetic field and an axial through flow with a suitable strength (typically for small strength) is stabilizing basic states. In general, circular Couette flow (*CCF*), (wavy) Taylor vortex flow [$(w)TVF$] and (wavy) spiral vortices [$(w)SPI$] can occur at a specific Reynolds number (Taylor number) without applying any axial through flow^32–35^ or magnetic fields^18–20,26,36^. But, when an axial through flow or a magnetic field is applied to such flows, toroidal flow structures are translating with the axial flow, and a (transverse) magnetic field tends to hinder the motion (pinning effect) of the flow in the annulus^18,26,36^. Depending on a magnetic field strength, basic states at higher Reynolds number can be stabilized, or even at low Reynolds number, due to the large magnetic field strength, turbulence can be happened^23^. That is, a magnetic field has a tendency to shrink the range/interval of parameters related to the onset of primary instability towards turbulence. In the present study we demonstrate how much the interaction of the three different instabilities affects very complicated ferrofluid dynamics. Our main findings are the observation of *localized* wavy vortices ($wTV{F}_{l}$ and $wTV{F}_{l.{H}_{x}}$) and *previously unknown new modulated Mixed-Cross-Spirals* ($mMC{S}_{{H}_{x}}$) with three different dominant azimuthal wavenumbers.

## System Setting and Numerical Procedure

### Ferrofluidic Taylor-Couette System

We consider a standard Taylor-Couette System (TCS) consisting of two independently rotating cylinders. The inner and outer cylinders have radius *R*_*i*_ and *R*_*o*_, and they rotate with the angular speeds ${\omega }_{i}$ and ${\omega }_{o}$, respectively (in present study, outer cylinder is at rest, *i*.*e*., ${\omega }_{o}=0$). We fix the hight-to-gap aspect ratio $\Gamma \mathrm{=2}$ and the radius ratio of the cylinders, $b\equiv {R}_{i}/{R}_{o}=0.5$, which is typically used in experiments and corresponds to an axial wavenumber $k=3.14$. Within the annular gap between two concentric cylinders there is an incompressible, isothermal, homogeneous, mono-dispersed ferrofluid of kinematic viscosity *ν* and density $\rho$. Figure 1 shows a schematic picture of TCS. The boundary conditions at the cylinder surfaces are of the non-slip type, but axially periodic boundary condition over a period length $\Gamma$ is used. The system can be described in the cylindrical coordinate system $(r,\theta ,z)$ by the velocity field ${\boldsymbol{u}}=(u,v,w)$ and the corresponding vorticity $\nabla \times {\boldsymbol{u}}=(\xi ,\eta ,\zeta )$.

As homogeneous magnetic fields, a transversal field, ${\boldsymbol{H}}={H}_{x}{{\boldsymbol{e}}}_{x}$, and an axial field, ${\boldsymbol{H}}={H}_{z}{{\boldsymbol{e}}}_{z}$, are considered, where *H*_*x*_ (*H*_*z*_) indicates the field strength in the transverse (axial) direction, respectively. The gap width $d={R}_{o}-{R}_{i}$ and the diffusion time ${d}^{2}/\nu$ are chosen as the length and time scales of the system, respectively. The pressure is normalized by $\rho {\nu }^{2}/{d}^{2}$, and the magnetic field **H** and magnetization **M** can be normalized by the quantity $\sqrt{\rho /{\mu }_{0}}\nu /d$, where *μ*_0_ is the permeability of free space. These considerations lead to the following set of non-dimensionalized hydrodynamical equations^21,37^:

1 $$\begin{array}{rcl}({\partial }_{t}+{\bf{u}}\cdot \nabla ){\bf{u}}-{\nabla }^{2}{\bf{u}}+\nabla p & = & ({\bf{M}}\cdot \nabla ){\bf{H}}+\frac{1}{2}\nabla \times ({\bf{M}}\times {\bf{H}}),\\ \nabla \cdot {\bf{u}} & = & 0.\end{array}$$

For more detailed descriptions of these hydrodynamical equations, see **Methods**.

### External axial through flow *Re*

To enforce an external axial through flow throughout the annulus, we add a constant pressure gradient with size ${\partial }_{z}{p}_{{\rm{APF}}}$ to the axial velocity component in the ferrohydrodynamical equations. In the sub-critical regime (below the onset of any vortex structure), this pressure gradient forces an annular Poiseuille flow (*APF*)^38,39^. The radial profile of this axial through flow velocity is given by

2 $${w}_{{\rm{APF}}}(r)=\frac{{\partial }_{z}{p}_{{\rm{APF}}}}{4}[{r}^{2}+\frac{(1+b)\,\mathrm{ln}\,r}{(1-b)\,\mathrm{ln}\,b}+\frac{(1+b)\,\mathrm{ln}\,(1-b)}{(1-b)\,\mathrm{ln}\,b}-\frac{1}{{(1-b)}^{2}}].$$

Its mean value can be used to define the axial through flow Reynolds number

3 $${Re}:\,=\langle {w}_{{\rm{APF}}}(r)\rangle =-\,\frac{{\partial }_{z}{p}_{{\rm{APF}}}}{8}\frac{1-{b}^{2}+(1+{b}^{2})\,\mathrm{ln}\,b}{{(1-b)}^{2}\,\mathrm{ln}\,b},$$

which describes the externally applied additional axial pressure gradient. Therefore, a positive (negative) *Re* indicates an upward (downward) axial through flow, ${w}_{{\rm{APF}}}(r)$, in the positive (negative) *z* direction, respectively (see Fig. 1). It means that an axial through flow can be characterized by the Reynolds number *Re*, Eq. (3).

### Numerical method

The ferrohydrodynamical equations of motion can be solved^20,21,26^ by combining a standard, second-order finite-difference scheme in $(r,z)$ with a Fourier spectral decomposition in *θ* and (explicit) time splitting. The variables can be expressed as

4 $$f(r,\theta ,z,t)=\mathop{\sum }\limits_{m=-{m}_{{\rm{\max }}}}^{{m}_{{\rm{\max }}}}\,{f}_{m}(r,z,t)\,{e}^{im\theta },$$

where *f* denotes one of the variables $\{u,v,w,p\}$. For the parameter regimes considered, the choice ${m}_{{\rm{\max }}}=40$ provides adequate accuracy, which ensure to have at least the four largest azimuthal mode amplitudes. We use a uniform grid with spacing $\delta r=\delta z=0.02$ and time steps $\delta t < 1/3800$. These time steps were always well below the von Neumann stability criterion and by more than a factor of 3 below the Courant-Friederichs-Lewy criterion. The core structure of the here used code, G1D3^26^, without any terms describing ferrofluids and magnetic field interaction has been validated by using various control calculations with different *m*_*max*_ and/or grid spacings and comparison with either linear stability and/or experimental results^17^. Typical SPI frequencies have an error of less than about 0.2% and that typical velocity field amplitudes can be off by about 3–4% with good agreement with experimental spirals^17^. The current code emanated from this basic code has shown similar performance and accuracy regarding linear stability analysis^22,40,41^ and even more important has been proven correct in predictions as the non-rotating wavy vortices due to symmetry breaking transversal field, which has been experimentally confirmed afterwards^18^.

For diagnostic purposes we also evaluate the complex mode amplitudes ${f}_{m,n}(r,t)$ obtained from a Fourier decomposition in axial direction

5 $${f}_{m}(r,z,t)=\sum _{n}\,{f}_{m,n}(r,t)\,{e}^{inkz},$$

where $k=2\pi /\lambda$ is the wave number and $\lambda =\Gamma$ is the wavelength.

### Parameters setting and quantities

For the two fixed Reynolds number of the inner cylinder (${R}{{e}}_{i}=110$ or $R{e}_{i}=270$), the effects of the axial through flow will be investigated for each magnetic field. That is, for the fixed magnetic field setting (1. ${s}_{z}=0.6,\,{s}_{x}=0$ and 2. ${s}_{z}=0,\,{s}_{x}=0.6$), by varying the axial through flow $Re$, Eq. (3), dynamics of flow states will be investigated. Note that a magnetic field strength can be characterized by the Niklas parameter *s*_*N*_ (see Methods).

As a global measure for characterizing the flow state, we use the modal kinetic energy, *E*_*kin*_, defined by

6 $${E}_{kin}=\sum _{m}\,{E}_{m}=\frac{1}{2}\,{\int }_{0}^{2\pi }\,{\int }_{-\Gamma /2}^{\Gamma /2}\,{\int }_{{r}_{i}}^{{r}_{o}}\,{{\bf{u}}}_{m}{{\bf{u}}}_{m}^{\ast }r{\rm{d}}r{\rm{d}}z{\rm{d}}\theta ,$$

where **u**_*m*_ (${{\bf{u}}}_{m}^{\ast }$) is the *m*-th (complex conjugate) Fourier mode, Eq. (4), of the velocity field, respectively. We note that *E*_*kin*_ is constant (non-constant) for a steady (an unsteady) solution. For diagnostic purposes, we consider the time-averaged quantity (over one period *T*) ${\overline{E}}_{kin}={\int }_{0}^{T}\,{E}_{kin}{\rm{d}}t$ and the time-averaged mode amplitudes $|{\overline{u}}_{m,n}|$. Note that when time-averaged quantities are studied, a period time of a particular solution has been considered. The period time of a solution depends on parameters of a system, which is typically different for different flow structures. In addition, as a local measure to characterize the flow states, the azimuthal vorticity on the inner cylinder at symmetrically displaced two points on the mid-plane, ${\eta }_{\pm }=({r}_{i},0,\pm \,\Gamma /4,t)$, will be considered in the next section.

### Nomenclature

We focus on flow states in the wide-gap TCS (aspect ratio: $\Gamma =2$, axial wavenumber $k=3.14$) with a periodic domain under axial through flow, characterized by the Reynolds number *Re* [Eq. (3)], for applying either axial or transversal magnetic field, which is schematically shown in Fig. 1. Note that in our setting, toroidally closed Taylor vortex flow (*TVF*) and helical spiral vortex flow (spirals, *SPI*s) are not modified by an axial magnetic field. But, in the presence of a transverse magnetic field, all the flow states are *inherently three dimensional*^18,21,26^ with additional stimulated *m* ± 2 modes. That is, their flow states are wavy-like modulated, *i*.*e*., toroidally wavy vortex flows ($wTV{F}_{{H}_{x}}$) and helical wavy spirals ($wSPI{s}_{{H}_{x}}$). All abbreviations used in the manuscript are listed in Table 1 including a short description of their main characteristics/properties.

**Table 1:** Nomenclature and abbreviations of flow states.

Abbreviation	Flow state & description
*CCF*	Circular Couette flow
*TVF*	Taylor vortex flow
*m*-*wTVF*	wavy Taylor vortex flow^46^ with major azimuthal wavenumber *m* (wTVF is also known as wavy vortex flow, *WVF*^?^)
*m*-$wTV{F}_{l}$	(axial) localized *m*-*wTVF*
*m*-$wTV{F}_{{H}_{x}}$	*m*-*wTVF* modified due to transverse magnetic field^26^ with *m* ± 2 modes
*m*-$wTV{F}_{l,{H}_{x}}$	(axial) localized *m*-$wTV{F}_{{H}_{x}}$
*m*_1_-*m*_2_-$wTV{F}_{{H}_{x}}$	$wTV{F}_{{H}_{x}}$ with major azimuthal wavenumber *m*_1_ and minor azimuthal wavenumber *m*_2_
L[R]*m*-*SPI*	left- [right-] winding spiral vortex flow with azimuthal wavenumber *m*^47^
*m*-*wSPI*	wavy *SPI* with major azimuthal wavenumber *m*^46^
L[R]*m*-$wSP{I}_{{H}_{x}}$	left- [right-] modified winding *m*-*wSPI* with *m* ± 2 modes due to transverse magnetic field^26^
L*m*_1_L*m*_2_-*MCS*	left-winding Mixed-Cross-Spiral^47^ with major azimuthal wavenumber *m*_1_ and minor azimuthal *m*_2_ (right-winding counterparts exist simultaneously)
L*m*_1_L*m*_2_-$MC{S}_{{H}_{x}}$	modified L*m*_1_L*m*_2_-*MCS* with *m* ± 2 modes due to transverse magnetic field
L*m*_1_L*m*_2_L*m*_3_-$mMC{S}_{{H}_{x}}$	left-winding modulated $MC{S}_{{H}_{x}}$ with a combination of azimuthal wavenumbers *m*_1_, *m*_2_ and *m*_3_

In the presence of a transverse magnetic field (${s}_{x}\ne 0$), all flow state are inherently 3D and have stimulated 2-fold symmetry^20,26^, which are indicated by a small label *H*_*x*_. The index *l* highlights the (axial) localization of the wavy flow structure.

### Bifurcation with an inner Reynolds number *Re*_*i*_ and *Re* = 0

Before investigating effects of imposing axial through flow, by varying an inner Reynolds number *Re*_*i*_, we first examine states of flow for each fixed magnetic field in the *absence* of such axial through flow, *i*.*e*., *Re* = 0.

**Figure 2:**
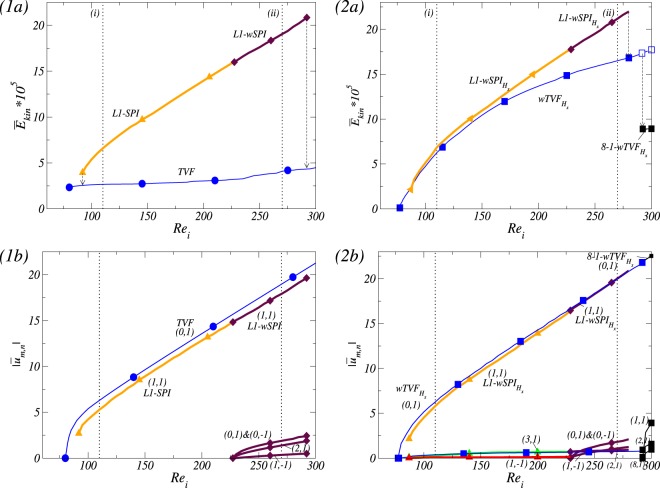
Bifurcation scenarios with $R{e}_{i}$ for axial and transversal magnetic field. Bifurcation scenarios with the Reynolds number $R{e}_{i}$ of the inner cylinder rotation (outer cylinder at rest) in (1) axial magnetic field and (2) transverse magnetic field, respectively. Shown are (**a**) the total (time-averaged for unsteady flow solutions) modal kinetic energy ${\overline{E}}_{kin}$ and (**b**) the corresponding dominant (time-averaged) mode amplitudes $|{\overline{u}}_{m,n}|$ of the radial velocity field at mid-gap as indicated [in notation $(m,n)$]. Different flow structures are labeled and color coded symbols are used in order to identify the structures. Vertical arrows indicate a transition behavior when a flow state loses stability and change to another stable state. Note that the mirror symmetric flow structures of right-winding spirals, *R*1-*SPI* and *R*1-$wSP{I}_{{H}_{x}}$, exist simultaneously. Two vertical dotted lines indicate $R{e}_{i}=110$ and $R{e}_{i}=270$, respectively.

By increasing *Re*_*i*_, Fig. 2(1 and 2) show the corresponding bifurcation scenario for two fixed magnetic fields, axial (${s}_{z}=0.6$) and transverse (${s}_{x}=0.6$) magnetic fields, respectively. Qualitative change of flow states shown in Fig. 2 is similar to the classical result of primary and secondary instabilities, appearing via supercritical Hopf bifurcations^1,2,42–44^. However, there are two *crucial differences*: (1) the critical values ${R}{{e}}_{i,c}$ (onset of instabilities) are *shifted* towards larger critical values due to the stabilizing effect of any magnetic field on the basic state^18,26,45^, and (2) due to a symmetry breaking effect of a transverse magnetic field, all flow states are intrinsically *three-dimensional*^18,20,21,26,36^. That is, in the presence of a transverse magnetic field, states of flow are *wavy modulated* solutions with additional stimulated $m\pm 2$ modes [Fig. 2(2)].

In detail, for an applied axial [transverse] magnetic field, Circular Couette flow (CCF) [2-fold annular vortex flow^1^ (2-AVF)] as a basic state is disrupted by a rotational instability and *stable* Taylor vortices (*TVF*) [wavy Taylor vortices ($wTV{F}_{{H}_{x}}$)] appeared in a supercritical primary bifurcation, and for larger values *Re*_*i*_, *unstable* spiral vortices (*SPI*) [wavy spiral vortices ($wSP{I}_{{H}_{x}}$)] bifurcate likewise supercritical primary out off CCF [2-AVF], respectively. By increasing *Re*_*i*_, these helical flows are stabilized, and then for more larger *Re*_*i*_, a system eventually become bistable. For an axial or a transverse magnetic field, the helical flow states are destabilized by a new wavy solution *w*-*SPI* [$wSP{I}_{{H}_{x}}$] which significantly contributes the emergence of the axisymmetric mode $m=0$. These flows finally lose their stability and transient towards the only remaining stable solution, *wTVF* for an axial field and $wTV{F}_{{H}_{x}}$ for transverse field, respectively. For a transverse magnetic field, $wTV{F}_{{H}_{x}}$ destabilizes against a localized wavy flow state, 8-1-$wTV{F}_{l,{H}_{x}}$.

Note that the bifurcation diagrams for 1$|{\overline{u}}_{m,n}|$ only show the dominant mode amplitudes which are incorporated in the flow structures. Further modes are also finite, but significant smaller and do not (or at least minor) contribute to the flow structure and dynamics. Vertical arrows in the energy plots indicate the transient scenario of a flow state. Note that in absence of an axial through flow, the symmetry related structures of right-winding and left-winding $[w]SP{I}_{{H}_{x}}$ are simultaneously existing.

## Results

To investigate the effects of an externally imposed axial through flow *Re*, for a fixed magnetic field, we will examine dynamics of flow states by varying *Re*, specially focusing on bifurcation phenomenon for two fixed inner Reynolds numbers (${R}{{e}}_{i}=110,270$).

### Effects of an axial through flow *Re* under applying only a fixed transversal magnetic field (*s*_*x*_ = 0.6)

**Figure 3:**
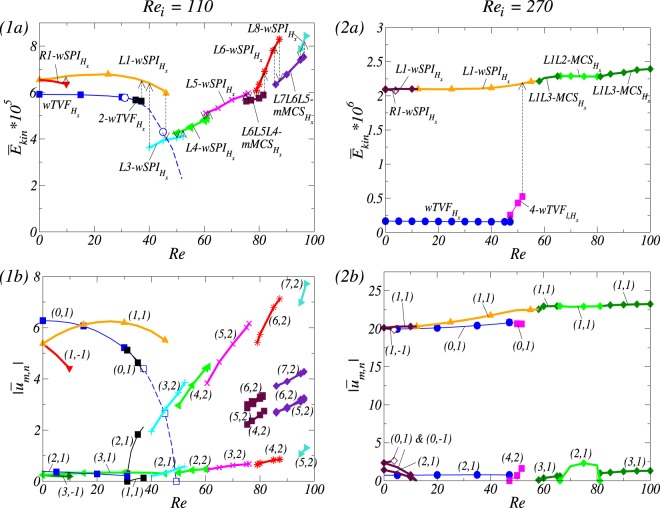
For applying the only transversal magnetic field ${s}_{x}=0.6$, Bifurcation scenarios vs. an imposed axial through flow *Re*. Bifurcation scenarios for (1) $R{e}_{i}=110$ and (2) $R{e}_{i}=270$, respectively. Shown are (**a**) the total (time-averaged) modal kinetic energy ${\overline{E}}_{kin}$ and (**b**) the corresponding dominant (time-averaged) amplitudes $|{\overline{u}}_{m,n}|$ of the radial velocity field at mid-gap as indicated [in notation $(m,n)$]. Different flow structures are labeled [in (1)] and same color code and symbols are used in (**a** and **b**) to identify the structures. Vertical arrows in (**a**) illustrate the transition direction to another stable state, when a flow state loses its stability. Note that thin [thick] lines correspond to toroidally closed [helical] flow states. Same legends are used for all kind of bifurcation sequences of different flow states throughout the paper.

For ${R}{{e}}_{i}=110$, the system at $Re=0$ shows *multistability* having three stable states, *L*1-$wSP{I}_{{H}_{x}}$, *R*1-$wSP{I}_{{H}_{x}}$ and $wTV{F}_{{H}_{x}}$ [see Fig. 2(2a)]. Note that by applying/increasing *Re*, two flow states *R*1-$wSP{I}_{{H}_{x}}$ and $wTV{F}_{{H}_{x}}$ move to *L*1-$wSP{I}_{{H}_{x}}$, and then follow the destiny of *L*1-$wSP{I}_{{H}_{x}}$. Figure 3 presents the variation of (time-averaged) modal kinetic energy ${\overline{E}}_{kin}$ of flow states and its corresponding dominant (time-averaged) mode amplitudes $|{\overline{u}}_{m,n}|$ for different Reynolds number of the inner cylinder. The dominant mode amplitudes $|{\overline{u}}_{m,n}|$ shown in Fig. 3 are incorporated in the flow structures. Thus, other modes might be finite but significant smaller, which only minorly contribute to the flow structure and dynamics. We note that vertical arrows in the energy plots [Fig. 3(1a and 2a)] indicate a transient behavior due to the change of its stability. Due to a transversal magnetic field, all flow states are inherently 3*D* and wavy-like modulated flow^26^ containing specific higher modes $m\pm 2$ [see Fig. 3(1b and 2b)].

The state $wTV{F}_{{H}_{x}}$ becomes unstable at $Re\approx 35$, and then bifurcates to a wavy flow state 2-$wTV{F}_{{H}_{x}}$ with dominant mode amplitudes $(2,\pm \,1)$. At $Re\approx 38$, 2-$wTV{F}_{{H}_{x}}$ finally loses its stability, and then towards a modulated helical spiral state *L*1-$wSP{I}_{{H}_{x}}$ [see Fig. 3(1a)]:

$$wTV{F}_{{H}_{x}}\to 2 \mbox{-} wTV{F}_{{H}_{x}}\to L1 \mbox{-} wSP{I}_{{H}_{x}}.$$

The downward propagating state *R*1-$wSP{I}_{{H}_{x}}$ becomes unstable at $Re\approx 10$, and then moves toward a stable upward propagating state *L*1-$wSP{I}_{{H}_{x}}$:

$$R1 \mbox{-} wSP{I}_{{H}_{x}}\to L1 \mbox{-} wSP{I}_{{H}_{x}}.$$

Starting with *L*1-$wSP{I}_{{H}_{x}}$, we obtain the following bifurcation sequence, as shown in Fig. 3(1a):

$$\begin{array}{l}L1 \mbox{-} wSP{I}_{{H}_{x}}\to L3 \mbox{-} wSP{I}_{{H}_{x}}\to L4 \mbox{-} wSP{I}_{{H}_{x}}\to L5 \mbox{-} wSP{I}_{{H}_{x}}\\ \,\to L6L5L4 \mbox{-} mMC{S}_{{H}_{x}}\to L6 \mbox{-} wSP{I}_{{H}_{x}}\to L7L6L5 \mbox{-} mMC{S}_{{H}_{x}}\to L8 \mbox{-} wSP{I}_{{H}_{x}}.\end{array}$$

From this bifurcation, we finally find a **new** type of *mixed mode states* called **modulated Mixed-Cross-Spirals** ($mMC{S}_{{H}_{x}}$) with *three* dominant azimuthal wavenumbers. For instance, *L*6*L*5*L*4-$mMC{S}_{{H}_{x}}$ and *L*7*L*6*L*5-$mMC{S}_{{H}_{x}}$.

The interesting thing is that $mMC{S}_{{H}_{x}}$ can be found between two spiral vortex flows with different wavenumbers. To understand a mode type of the newly found $mMC{S}_{{H}_{x}}$, for instance *L*6*L*5*L*4-$mMC{S}_{{H}_{x}}$ which is appeared as a *stable* state between two stable states, *L*6-$wSP{I}_{{H}_{x}}$ and *L*5-$wSP{I}_{{H}_{x}}$, [see Fig. 3(1b)], one might intuitively expect that the mixed structure is just a combination of the two dominant azimuthal wavenumbers, $m=5$ and $m=6$. But, it is not true! Actually, the mixed mode of *L*6*L*5*L*4-$mMC{S}_{{H}_{x}}$ consists of ***three*** dominant azimuthal wavenumbers, $m=4$, 5 and 6, which is *another result* of the symmetry breaking effect induced by the transverse magnetic field. For the dominant modes ($m=4$, 5 and 6) of *L*6*L*5*L*4-$mMC{S}_{{H}_{x}}$, $m=5$ and 6 are induced from *L*5-$wSP{I}_{{H}_{x}}$ and *L*6-$wSP{I}_{{H}_{x}}$, respectively, but $m=4$ from the additionally stimulated finite mode $m=4$ within *L*6-$wSPI$ which is the stimulated $m\pm 2$ mode of a transverse magnetic field. It will be explained in the next section.

For $R{e}_{i}=270$, in the absence of an axial through flow (${Re}=0$), we may observe *L*1-$wSP{I}_{{H}_{x}}$, *R*1-$wSP{I}_{{H}_{x}}$ and $wTV{F}_{{H}_{x}}$ as multistable states, which is shown the previous paragraph. The helical state *L*1-$wSP{I}_{{H}_{x}}$ bifurcates in the following way [see Fig. 3(2a)]:

$$L1 \mbox{-} wSP{I}_{{H}_{x}}\to L1L3 \mbox{-} MC{S}_{{H}_{x}}\to L1L2 \mbox{-} MC{S}_{{H}_{x}}\to L1L3 \mbox{-} MC{S}_{{H}_{x}}.$$

At $Re\approx 58.3$, *L*1-$wSP{I}_{{H}_{x}}$ bifurcates into the Mixed-Cross-Spiral state *L*1*L*3-$MC{S}_{{H}_{x}}$. By more increasing the axial through flow *Re*, the first appeared *MCS* state will bifurcate into a different types of *MCS*, *i*.*e*., *L*1*L*3-$MC{S}_{{H}_{x}}$ $\to$ *L*1*L*2-$MC{S}_{{H}_{x}}$ at $Re\approx 65.8$, and then at $Re\approx 81.4$, it returns back the former one, *i*.*e*., *L*1*L*2-$MC{S}_{{H}_{x}}$ $\to$ *L*1*L*3-$MC{S}_{{H}_{x}}$ as shown in Fig. 3(2a).

We note that *R*1-$wSP{I}_{{H}_{x}}$ will be become unstable at $Re\approx 10$, and then evolve into *L*1-$wSP{I}_{{H}_{x}}$:

$$R1 \mbox{-} wSP{I}_{{H}_{x}}\to L1 \mbox{-} wSP{I}_{{H}_{x}}.$$

Figure 3(2b) shows the bifurcation sequence of the state $wTV{F}_{{H}_{x}}$:

$$wTV{F}_{{H}_{x}}\to 4 \mbox{-} wTV{F}_{l,{H}_{x}}\to L1 \mbox{-} wSP{I}_{{H}_{x}}.$$

We observe that at $Re\approx 47.6$, $wTV{F}_{{H}_{x}}$ bifurcates to 4-$wTV{F}_{l,{H}_{x}}$ with dominant azimuthal wavenumber $m=4$. Actually, 2-$wTV{F}_{l,{H}_{x}}$ and 3-$wTV{F}_{l,{H}_{x}}$ as only transient states can be temporarily observed. At $Re\approx 52$, 4-$wTV{F}_{l,{H}_{x}}$ loses its stability, and then moves towards the helical *L*1-$wSP{I}_{{H}_{x}}$. Note that through this bifurcating process, we may more detect 5-$wTV{F}_{l,{H}_{x}}$ and 6-$wTV{F}_{l,{H}_{x}}$ as a transient state. Interestingly we could not observe any other stable $wTV{F}_{l,{H}_{x}}$ with larger azimuthal wavenumber.

**Figure 4:**
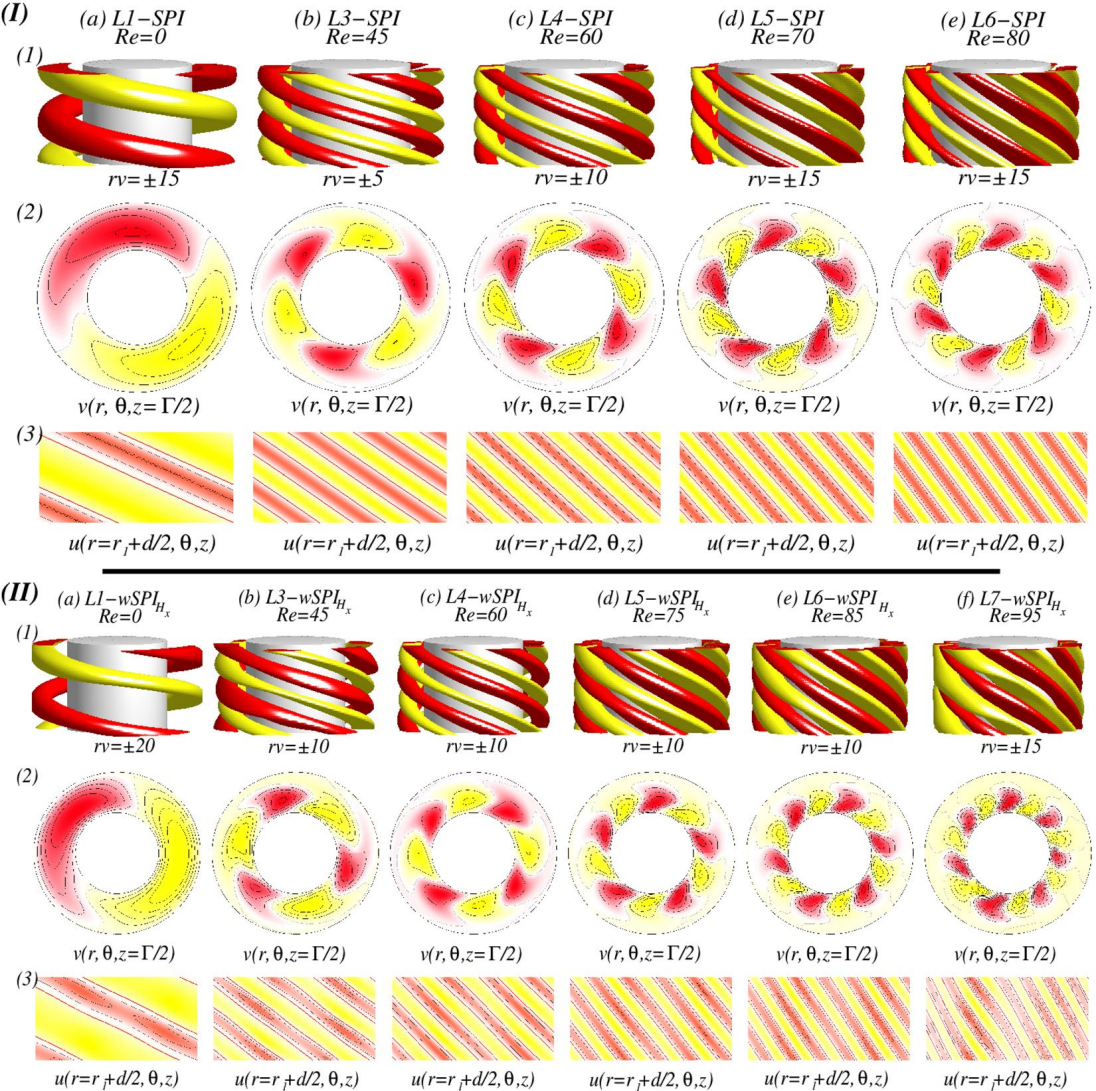
Flow visualizations with *Re* under the influence of a transversal magnetic field ${s}_{x}=0.6$. (**I**) and (**II**) Show flow visualization of a helical flow state $wSP{I}_{{H}_{x}}$ for different Reynolds number $R{e}_{i}=100$ and 270, respectively. Shown are (1) isosurface of *rv*; (2) the azimuthal velocity $v(r,\theta ,z=\Gamma /2)$ at mid-plane; and (3) the radial velocity $u(r={r}_{i}+d/2,\theta ,z)$ at mid-gap. Red (dark gray) and yellow (light gray) correspond to positive and negative values, respectively, with zero specified as white. The same legends are used for all visualizations of different flow states in the paper.

For various *Re*, visualization of a helical flow $wSP{I}_{{H}_{x}}$ for different Reynolds number *Re*_*i*_ can be seen in Fig. 4. The dominant azimuthal wavenumber *m* is obviously increased with *Re*. The additional *m* ± 2 modes induced by a symmetry breaking effect of a transversal magnetic field can be best seen in wavy modulated spiral flows as shown in Fig. 4 (see iso-surfaces and radial velocity in Fig. 4). By increasing *Re*, the flow structures with higher azimuthal wavenumber *m* are close located towards the inner cylinder [see Fig. 4]. It means that the outer bulk region becomes almost vortex free. Thus in *mMCS*, the dominant modes decrease through the bulk, from inside to outside. In the case of *L*6*L*5*L*4-*mMCS*, it means a sequence of azimuthal wavenumbers *m*: $6\to 5\to 4$ [Fig. 4(4)].

### Modulated Mixed-Cross-Spirals (${\boldsymbol{mMC}}{{\boldsymbol{S}}}_{{{\boldsymbol{H}}}_{{\boldsymbol{x}}}}$)

**Figure 5:**
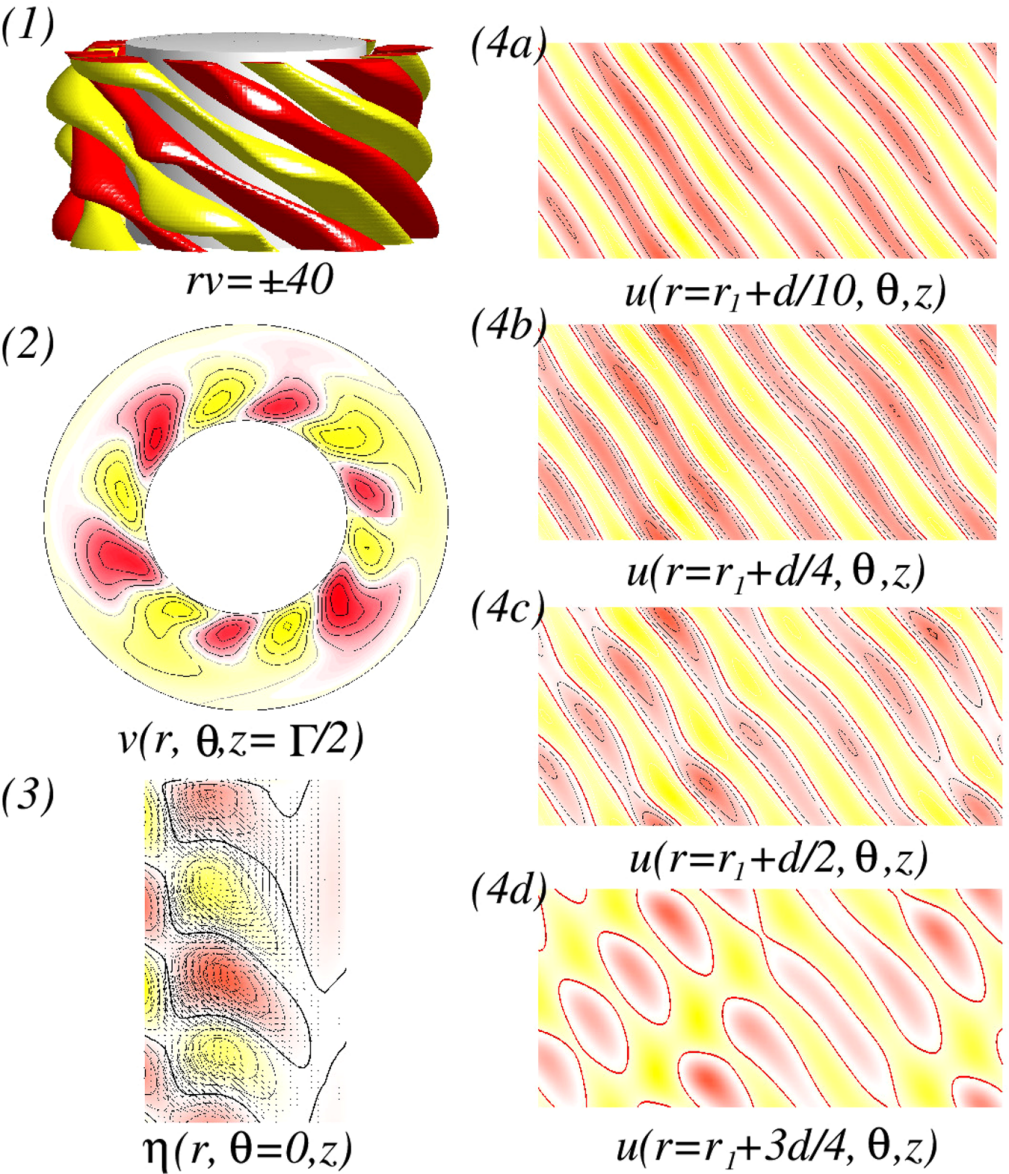
Flow visualizations of *L*6*L*5*L*4-$mMC{S}_{{H}_{x}}$. Flow visualization of flow state *L*6*L*5*L*4-$mMC{S}_{{H}_{x}}$ with ${s}_{x}=0.6$, $R{e}_{i}=110$ and $Re=82$. Presented are (1) isosurface of *rv*; (2) the azimuthal velocity $v(r,\theta )$ at mid-plane ($z=\Gamma /2$); and (3) vector plot $[u(r,z),w(r,z)]$ of the radial (*u*) and axial (*w*) velocity components including color-coded azimuthal vorticity $\eta (r,\theta =0,z)$). (4a–4d) Show the radial velocity $u(r,\theta ,z)$ at different radial positions as indicated (See SM movie 1).

Now, at $R{e}_{i}=110$, the newly detected $mMC{S}_{{H}_{x}}$ with mixed mode structures will be investigated, which can be found in *only* between two $wSP{I}_{{H}_{x}}$ states in the presence of a transverse magnetic field. Figure 5 shows the spatial structure of a *stable L*6*L*5*L*4-$mMC{S}_{{H}_{x}}$ which exists between *L*5-$wSP{I}_{{H}_{x}}$ and *L*6-$wSP{I}_{{H}_{x}}$. From Fig. 5, we may clearly see the dominance of the mode $m=6$, and also from the different plots of unrolled cylinders surfaces $u(r,\theta ,z)$ presented in Fig. 5(4), the influence and modulation of both other dominant modes $m=5$ and $m=4$ could be highlighted. The dominant azimuthal wavenumber *m* is decreasing from the inner towards the outer cylinder.

**Figure 6:**
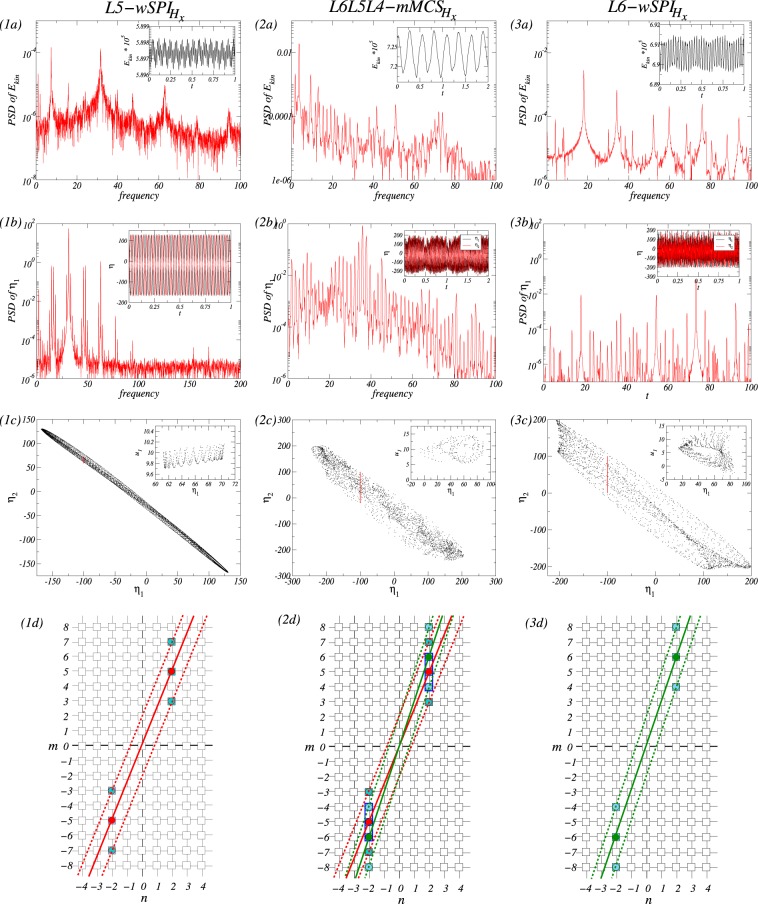
Comparison of $mMC{S}_{{H}_{x}}$ and $wSP{I}_{{H}_{x}}$: For ${s}_{x}=0.6$, (**a**–**c**) show various quantities of flow states, *L*5-$wSP{I}_{{H}_{x}}$ at $Re=75$, *L*6*L*5*L*4-$mMC{S}_{{H}_{x}}$ at $Re=82$ and *L*6-$wSP{I}_{{H}_{x}}$ at $Re=85$, respectively. Shown are power spectral density (PSD) of (**a**) ${E}_{kin}$ and (**b**) ${\eta }_{1}$ (${\eta }_{2}$), where ${\eta }_{1[2]}=\eta ({r}_{i},\theta ,\Gamma /8[3\Gamma /8],t)$; Insets in (**a**,**b**) show corresponding time series. (**c**) Shows phase portraits in the $({\eta }_{1},{\eta }_{2})$ plane. The inset in (**c**) shows the corresponding two-dimensional Poincaré section $({\eta }_{1},{u}_{1})$, where ${u}_{1}=u({r}_{i},\theta ,\Gamma /8,t)$, at ${\eta }_{1}=-\,100$ for $\theta =0$ (indicated by red line). (**d**) Presents dominant excited modes (colored squares) of the different solutions in the two-dimensional Fourier mode space spanned by the azimuthal and axial Fourier mode indices *m* and *n*. Filled circles denote linearly driven modes and linear Fourier mode subspaces are indicated by thick lines. They represent *L*5-$wSP{I}_{{H}_{x}}\mathrm{(5}n\pm \mathrm{2,}n)$, *L*6-$wSP{I}_{{H}_{x}}\mathrm{(6}n\pm \mathrm{2,}n)$, and a combination of both in *L*6*L*5*L*4-$mMC{S}_{{H}_{x}}$.

For a more detailed quantitative analysis of $mMC{S}_{{H}_{x}}$, we calculate power spectral densities (PSDs) and time series of the global quantity *E*_*kin*_ as well as the local quantities ${\eta }_{\pm }$ of flow states *L*5-$wSP{I}_{{H}_{x}}$, *L*6-$wSP{I}_{{H}_{x}}$ and *L*6*L*5*L*4-$mMC{S}_{{H}_{x}}$, respectively. Figure 6(1) shows the time series of the modal kinetic energy *E*_*kin*_ and ${\eta }_{\pm }$ together with its corresponding power spectral densities (PSDs) for these states, which show complex dynamics incorporating various frequencies. From PSDs of *E*_*kin*_, we may observe that the dominant frequency of *L*5-$wSP{I}_{{H}_{x}}$ (*L*6-$wSP{I}_{{H}_{x}}$) is ${\omega }_{L5w}\approx 31.62$ (${\omega }_{L6w}\approx 18.09$), respectively [see Fig. 6(1a and 3a)]. Due to the azimuthal drift of the flow structure itself induced by a combination of a transversal magnetic field and an axial-through flow, complicated dynamics incorporating all linear combinations of $wSP{I}_{{H}_{x}}$ can be seen through the local quantities ${\eta }_{1}$ and ${\eta }_{2}$ [see Fig. 6(b)]. From the phase-space projections and Poincaré sections shown in Fig. 6(c), *L*5-$wSP{I}_{{H}_{x}}$ and *L*6-$wSP{I}_{{H}_{x}}$ may be considered to live on a 2-torus with an additional drift frequency. Like chaotic behavior, a complexity of time series and PSDs as shown in Fig. 6(2b) suggests that $mMC{S}_{{H}_{x}}$ may live on 3-tori invariant manifolds.

To understand the mixed modes of $mMC{S}_{{H}_{x}}$, for instance, we will deal with *L*6*L*5*L*4-$mMC{S}_{{H}_{x}}$. Due to the symmetry-breaking effect of a transversal magnetic field, the helical state *L*5-$wSP{I}_{{H}_{x}}$ [*L*6-$wSP{I}_{{H}_{x}}$] has its dominant mode $m=5$ [$m=6$] and an additionally stimulated modes $m\pm 2=\{3,7\}$ [$m\pm 2=\{4,8\}$]^26^, respectively. When two states *L*5-$wSP{I}_{{H}_{x}}$ and *L*6-$wSP{I}_{{H}_{x}}$ come *close* in *Re* regime, they stimulate one of these additional modes (here $m=4$) as a nonlinear interaction of dominant modes. Finally, a new state with three dominant modes is created. To see it, Fig. 6(4) shows the dominant excited modes (colored squares) of flow states in the two-dimensional Fourier mode space $(m,n)$ spanned by the azimuthal and axial Fourier modes. The filled circles denote linearly driven modes, and then thick lines as linear Fourier mode subspaces are superimposed on them. A mode space of *L*6*L*5*L*4-$mMC{S}_{{H}_{x}}$ can be constituted as a combination of two mode spaces of *L*5-$wSP{I}_{{H}_{x}}$ and *L*6-$wSP{I}_{{H}_{x}}$ [$(5n\pm 2,n)$ and $(6n\pm 2,n)$].

To more detect stable $mMC{S}_{{H}_{x}}$ at $R{e}_{i}=110$, we performed further simulations between other two $wSP{I}_{{H}_{x}}$ with different azimuthal wavenumber, and then finally find a stable *L*7*L*6*L*5-$mMC{S}_{{H}_{x}}$ between two stable states *L*7-$wSP{I}_{{H}_{x}}$ and *L*6-$wSP{I}_{{H}_{x}}$ [see Fig. 3(2b)]. But, in lower *Re* regimes, despite many efforts, any search of further $mMC{S}_{{H}_{x}}$ remained unsuccessful. One of reasons we could not detect might be a bistability of flow states with lower azimuthal wavenumber. For instance, between *L*4-$wSP{I}_{{H}_{x}}$ and *L*3-$wSP{I}_{{H}_{x}}$, and between *L*5-$wSP{I}_{{H}_{x}}$ and *L*4-$wSP{I}_{{H}_{x}}$ [see Fig. 3(2b)], we could not observe more stable state $mMC{S}_{{H}_{x}}$. However, we may detect unstable states $mMC{S}_{{H}_{x}}$ to be appeared in a transient manner. For instance, *L*4*L*3*L*2-$mMC{S}_{{H}_{x}}$ can be observed as an unstable state in the transient process of between two stable *L*4-$wSP{I}_{{H}_{x}}$ and *L*3-$wSP{I}_{{H}_{x}}$ at lower *Re*.

### Effects of an axial-through flow *Re* under applying only a fixed axial magnetic field (*s*_*z*_ = 0.6)

**Figure 7:**
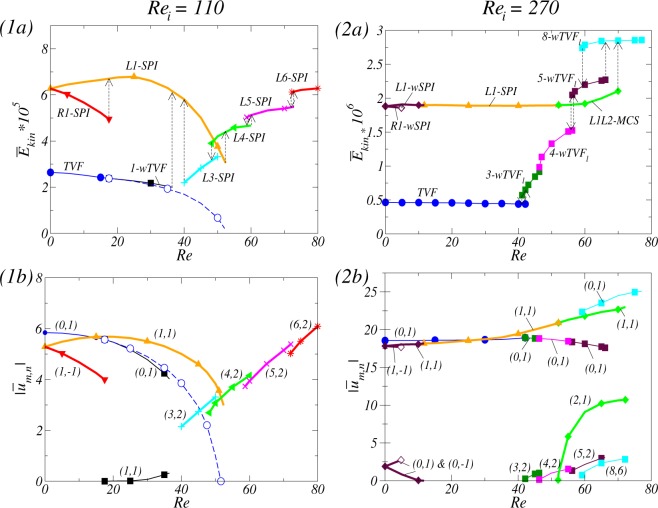
For applying the only axial magnetic field ${s}_{z}=0.6$, Bifurcation scenarios vs. an imposed axial through flow *Re*. As Fig. 3 but for the different magnetic field. Different flow structures are labeled in (1).

For $R{e}_{i}=110$ and in the absence of an axial through flow ($Re=0$), the system shows *multistabilty*. That is, there exist three stable flow states: *L*1-*SPI* and *R*1-*SPI* (which are degenerated by symmetry), and *TVF* [see Fig. 2(1)]. By applying/increasing *Re*, the helical spiral state *L*1-*SPI* will bifurcate the following way [see Fig. 7(1a)]:

7 $$L1 \mbox{-} SPI\to L4 \mbox{-} SPI\to L5 \mbox{-} SPI\to L6 \mbox{-} SPI.$$

In this bifurcation sequence, the increase of their azimuthal wavenumber *m* can be observed in a relatively small *Re* regime. In general, for larger values of an imposed axial through flow *Re*, flow states with larger azimuthal wavenumber *m* can be observed^17^. At $Re\approx 53$, *L*1-*SPI* directly moves to *L*4-*SPI*. Thus, we could not observe stable *L*2-*SPI* with dominant $m=2$. But, we could not say that *L*2-*SPI* does not exist, because there are some possibilities that (1) such state maybe only exist as unstable or (2) a region of stability might be too narrow or *far* away from other stable states. Therefore, in our numerical simulation, this state can not be detected as a stable state. However, when *Re* is decreased [see Fig. 7(1a)], *L*3-*SPI* can be found, which shows the existence of flow states with azimuthal wavenumber $m=3$.

The initially existing downward propagating state $R1-SPI$ exists as stable only for relatively small value *Re*. But, for a suitable axial-through flow, it immediately loses its stability, and then moves towards a stable upward propagating state *L*1-*SPI* [see Fig. 7(1a)]:

$$R1 \mbox{-} SPI\to L1 \mbox{-} SPI.$$

The upward propagating structure can not be resisted by a strong headwind force which is generated by an artificially and oppositely applied axial through flow in their propagation. That is, the applied axial through flow ${Re} > 0$ (downward from top to bottom) is working on destroying the natural propagating direction of *R*1-*SPI*. Note that the symmetry-related flow states of right-winding identically exist for an oppositely directed axial trough flow, −*Re*.

The *TVF* state with toroidally closed structure loses its stability at $Re\approx 15$, and then bifurcates into 1-*wTVF*^46^ with dominant mode amplitudes $(1,\pm 1)$. For larger $Re(\,\approx \,\mathrm{35)}$, 1-*wTVF* finally moves towards a helical spiral state *L*1-*SPI*. Vertical arrows shown in Fig. 7(1a) indicate the bifurcation scenario:

$$TVF\to 1 \mbox{-} wTVF\to L1 \mbox{-} SPI.$$

When *Re* is decreasing from a large value (for instance, $Re=80$), the bifurcation sequence of *L*6-*SPI* can be observed in the following way [see Fig. 7(1)]:

8 $$L6 \mbox{-} SPI\to L5 \mbox{-} SPI\to L4 \mbox{-} SPI\to L3 \mbox{-} SPI\to L1 \mbox{-} SPI.$$

Here, we found the *hysteretic* effect [see Fig. 7(1a), and compare (7) and (8)]. Through this bifurcation sequence, we emphasize an observation of *L*3-*SPI*, which can not be observed when *Re* is increased. The finial destination of this sequence is not *TVF* but *L*1-*SPI*. It means that we could not recover *TVF* by decreasing *Re*. That is, the branches of solution are disconnected.

Note that in the absence of an axial through flow ($Re=0$), wavy modulated states can be observed for different *Re*_*i*_: *L*1-*wSPI* and *L*1-$wSP{I}_{{H}_{x}}$ for helical flow states and *TVF* and $wTV{F}_{{H}_{x}}$ for toroidally closed flow states^26^ (see Fig. 2). For a fixed larger Reynolds number $R{e}_{i}=270$, three different stable states *L*1-*wSPI*, *R*1-*wSPI* and *TVF* can be detected. By increasing *Re*, Fig. 7 presents its corresponding bifurcation diagram, which is in analogy to the plot shown in Fig. 3.

The bifurcation sequence of *L*1-*wSPI* can be observed in the following way [see Fig. 7(2a)]:

$$L1 \mbox{-} wSPI\to L1 \mbox{-} SPI\to L1L2 \mbox{-} MCS\to 8 \mbox{-} wTV{F}_{l}.$$

The initially existing *L*1-*wSPI* vanishes against a *L*1-*SPI* at $Re\approx 12.2$ (no more wavy-like modulation). At $Re\approx 52.2$, *L*1-*SPI* is disappeared, and then *L*1*L*2-*MCS* (Mixed-Cross-Spiral state) is born in a forward bifurcation. Here, its corresponding mode (2, 1) becomes finite [see Fig. 7(1b)]. Note that this secondary bifurcation from spirals to *MCS* is a supercritical forward Hopf bifurcation^47,48^. Further increasing *Re*, *L*1*L*2-*MCS* eventually loses its stability, and moves to a toroidally closed flow, 8-$wTV{F}_{l}$. The flow structure of 8-$wTV{F}_{l}$ is propagating downstream with *Re*.

Note that the mirror-symmetric state *R*1-*wSPI* loses its stability at $Re\approx 5$, and then bifurcates to *L*1-*wSPI* due to the headwind of an axial-through flow *Re* against its own natural propagation direction:

$$R1 \mbox{-} wSPI\to L1 \mbox{-} wSPI.$$

By increasing *Re*, a bifurcation sequence of *TVF* can be observed in the following way [see Fig. 7(2a)]:

$$TVF\to 3 \mbox{-} wTV{F}_{l}\to 4 \mbox{-} wTV{F}_{l}\to 5 \mbox{-} wTV{F}_{l}\to 8 \mbox{-} wTV{F}_{l}.$$

In detail, *TVF* loses its stability against a *localized* wavy flow state, 3-$wTV{F}_{l}$, with dominant azimuthal wavenumber $m=3$ at $Re\approx 42.3$, which is similar to the scenario of helical flow states *wSPI*, as shown in Fig. 7. By increasing further *Re*, as different localized states of $wTV{F}_{l}$, 4-$wTV{F}_{l}$, 5-$wTV{F}_{l}$ and 8-$wTV{F}_{l}$ with increase of their azimuthal wavenumber *m* can be observed. But, we could not observe 2-$wTV{F}_{l}$, 6-$wTV{F}_{l}$ and 7-$wTV{F}_{l}$, which is similar to the bifurcation scenarios of helical (wavy) spirals at $R{e}_{i}=110$ (see Figs 4 and 7). In fact, during 5-$wTV{F}_{l}$ bifurcates to 8-$wTV{F}_{l}$, we temporarily observe 6-$wTV{F}_{l}$ and 7-$wTV{F}_{l}$ as *transient flow states*. It means that these flow states are unstable. Similarly, we also temporarily observe 2-$wTV{F}_{l}$ as a *transient flow state*, when 3-$wTV{F}_{l}$ bifurcates to *TVF*.

When *Re* is decreasing from large value, *i*.*e*., our starting flow state is 8-$wTV{F}_{l}$, we see the following bifurcation sequence [see Fig. 7(2a)]:

$$8 \mbox{-} wTV{F}_{l}\to 5 \mbox{-} wTV{F}_{l}\to 4 \mbox{-} wTV{F}_{l}\to 3 \mbox{-} wTV{F}_{l}\to TVF\mathrm{.}$$

It implies that one can not find helical structures, but only *localized* wavy flow states.

**Figure 8:**
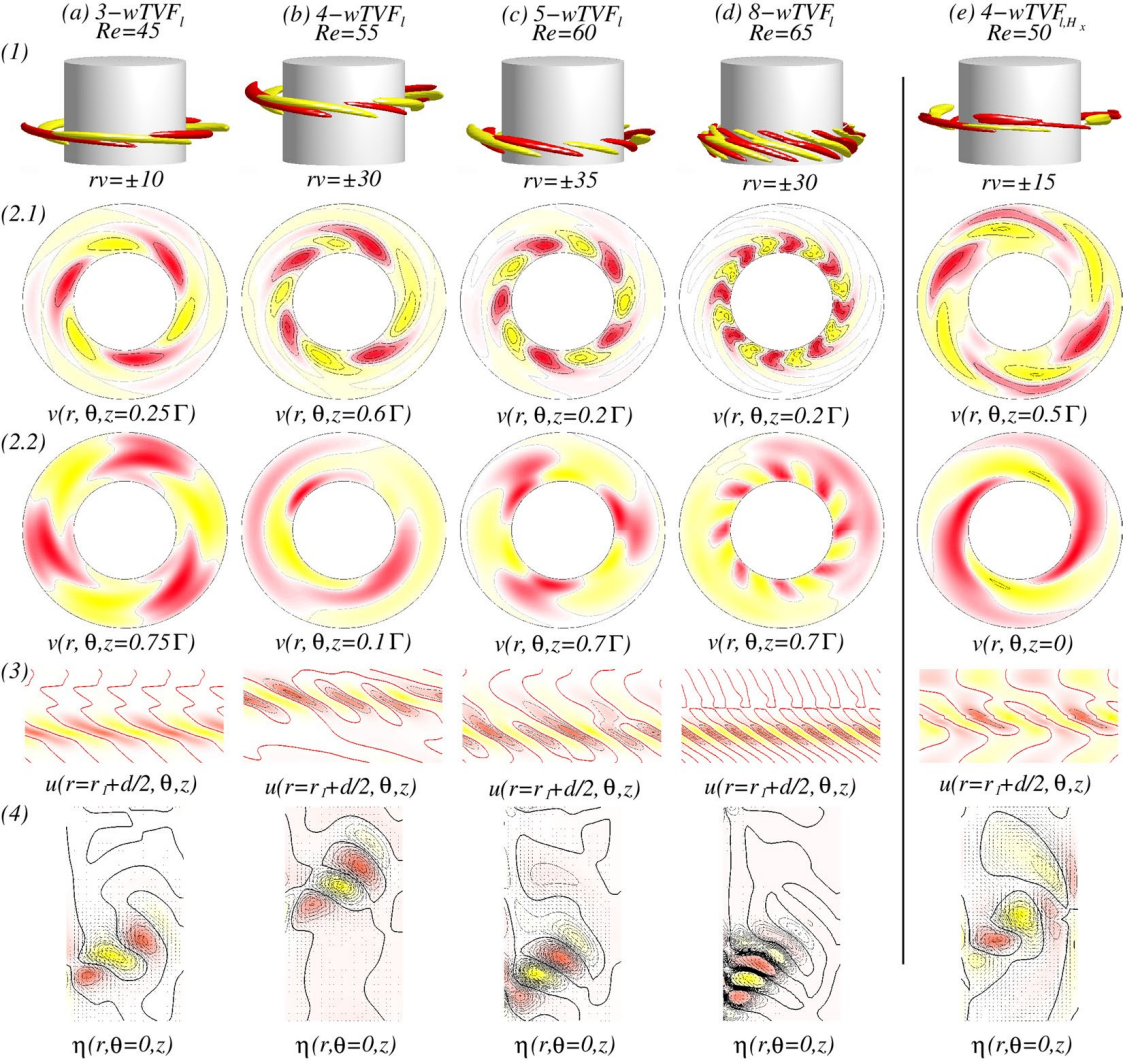
Flow visualizations with *Re* and $R{e}_{i}=270$. (**a**–**d**) Show flow visualization of toroidally closed, localized wavy flow structures in an axial field, ${s}_{z}=0.6$, and (**e**) for a transverse field. Flow structures and *Re* are indicated at the top. Shown are (1) isosurface of *rv*; (2) the azimuthal velocity $v(r,\theta ,z)$ within the localized wavy structure (2a) and $\Gamma /2$ away; (3) the radial velocity $u(r={r}_{i}+d/2,\theta ,z)$ at mid-gap; (4) vector plot $[u(r,z),w(r,z)]$ of the radial (*u*) and axial (*w*) velocity components including color-coded azimuthal vorticity $\eta (r,\theta =0,z)$). Red (dark gray) and yellow (light gray) colors correspond to positive and negative values, respectively, with zero specified as white. Note that all localized $wTV{F}_{{H}_{x}}$ travel upstream due the applied positive axial flow. With increasing *m*, Fig. 8(2.1) and (2.2) show the movement of flow structures towards the inner cylinder, and Fig. 8(4) shows more inclined situation for larger *Re* (See SM movies 2 & 3).

Figure 8 illustrates the different localized wavy states $wTV{F}_{l}$ ($wTV{F}_{l,{H}_{x}}$) in the presence of either an axial or a transverse magnetic field, respectively. It is worth to mention that all localized state $wTV{F}_{l,{H}_{x}}$ travel downstream due to the applied axial flow (from top to bottom). But, for a reversed axial through flow, −*Re*, flow states with the reverse direction equivalently exist. While the dominant azimuthal wavenumber is clear visible within the localized $wTV{F}_{l,{H}_{x}}$ [see Fig. 8(2.1)], there seems no direct connection between the secondary/background flow and these localized structures [Fig. 8(2.2)]. For instance, the backgrounds of 3-$wTV{F}_{l}$ [Fig. 8(a)], 5-$wTV{F}_{l}$ [Fig. 8(c)] and 8-$wTV{F}_{l}$ [Fig. 8(d)] show 3, 5, and 8-fold symmetry, respectively, but 4-$wTV{F}_{l}$ [Fig. 8(b)] does not show symmetry due to the domination of $m=1$ mode. However, we may observe that the background flows have equal or smaller azimuthal wavenumber, when compared to the localized structure, and helical state/structure $(w)SPI{s}_{{H}_{x}}$ itself moves towards the inner cylinder with increasing *m* and *Re*. That is, the main energy becomes stored within the localized flow structure.

In addition, the orientation of the localized $wTVF$ with larger azimuthal wavenumber *m* will be changed. At $Re=45$, the vortices of 3-*wTVF* are mainly radial orientated with certain moderate incline/slope [see Fig. 8(4a)], but for larger *Re*, it changes. That is, an orientation of 8-*wTVF* changes to a predominant axial orientation of *wTVF*. Even 4-$wTV{F}_{l,{H}_{x}}$ in the presence of a transverse magnetic field shows the same incline in radial direction, it is obviously wider in radial and axial directions, when compared to *m*-$wTV{F}_{l}$ in the axial field [compare Fig. 8(b,e) (2.1) and (4)]. The axial dimension/domain in which the localized $wTV{F}_{l,{H}_{x}}$ pattern exists remains almost the same for all detected localized flow states. Therefore, by increasing *Re*, as a result of enlarging the number of vortices with increasing the azimuthal wavenumber *m*, these vortices become more and more squeezed/compressed together [see Fig. 8(4)].

### Angular momentum transport

**Figure 9:**
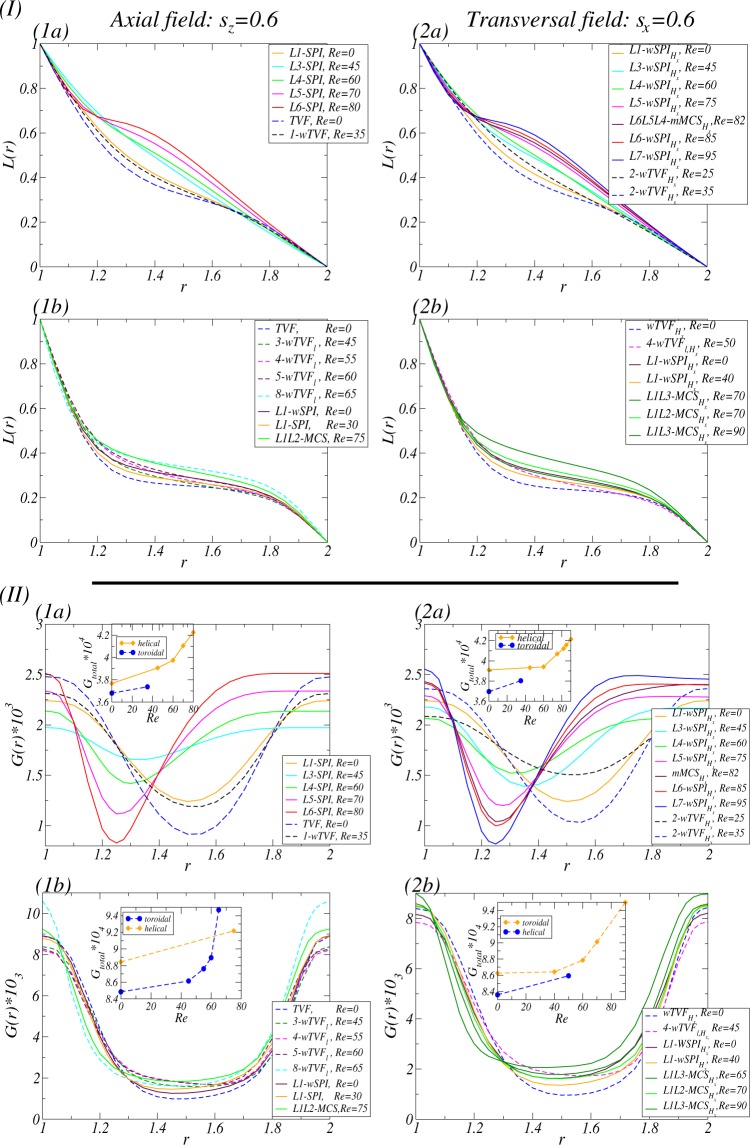
Variation in angular momentum and dimensionless torque. (**I**) Variation of angular momentum $L(r)=r{\langle v(r)\rangle }_{\theta ,z}/{R}{{e}}_{i}$ and (**II**) variation of the dimensionless torque $G=\nu {J}^{\omega }$ versus the radius *r* for solutions with increasing *Re* as indicated in axial and transverse magnetic field at (**a**) $R{e}_{i}=110$ and (**b**) $R{e}_{i}=270$. The insets in (**II**) show the variation of the total torque *G*_*total*_ for different *Re*.

To more characterize flow states, we examine an angular momentum and a torque for various flow structure. For a fixed *Re*_*i*_ and a magnetic field, Fig. 9(I) shows the mean profiles of axially or azimuthally averaged angular momentum $L(r)=r{\langle v(r)\rangle }_{\theta ,z}/R{e}_{i}$^23,49^ defined as a function of the radius *r*. In general, the profiles with positive angular momentum typically show a decrease from the rotating inner cylinder towards the stationary outer cylinder. Thus, all curves presented in Fig. 9(I) show similar shapes.

For $R{e}_{i}=110$ [see Fig. 9(I)–(1a) and (2a)], the profiles of the angular momentum $L(r)$ show a monotonically decreasing pattern. By increasing the azimuthal wavenumber *m* (which is correlated to *Re*), a general trend of the profiles for helical states is a change from a concave to a convex shape. In particular, a *belly* shape profile at a central region of *r* can be observed, and the maximum of these belly shape is increasing by *m*. Note that the newly found $mMC{S}_{{H}_{x}}$ states also follows these tendencies. But, for toroidally closed states, one can not observe any change in qualitative, instead of an increase of $L(r)$ at the center region in their absolute values. The key change in shape of the curves $L(r)$ can be highlighted by moving towards the inner cylinder or a dominant region.

For larger $R{e}_{i}=270$ [see Fig. 9(I)–(1b) and (2b)], the profiles of the angular momentum $L(r)$ show a more flatten pattern at the middle of the annular gap to form a horizontal plateau with nearly constant angular momentum, which is most pronounced for toroidal states. The angular momentum curves of helical states [$(w)SPI{s}_{{H}_{x}}$ or $MCS{s}_{{H}_{x}}$] show similar shapes. In general, the angular momentum curves follow a monotonically varying trend. By increasing the azimuthal wavenumber *m* of flow states (including toroidally closed and helical states), the central plateau-like region moves upwards to large values, and then becomes more incline. For $wTV{F}_{{H}_{x}}$ with larger *m*, the slope of $L(r)$ will be obviously increased due to the larger values of $L(r)$ at close to the inner cylinder. As already seen before, in general, the increase in the average angular momentum of the flow with larger azimuthal wavenumber *m* coincides with the pattern produced by larger values *Re*.

Figure 9(II) shows the corresponding variation of the dimensionless torque $G=\nu {J}^{\omega }$ within the annulus. In calculating the torque, we used the fact that for a flow between infinite cylinders the transverse current of the azimuthal motion, ${J}^{\omega }={r}^{3}[{\langle u\omega \rangle }_{A,t}-\nu {\langle {\partial }_{r}\omega \rangle }_{A,t}]$ (with ${\langle \ldots \rangle }_{A}\equiv \int \,\tfrac{rd\theta dz}{2\pi rl}$), is a conserved quantity^49^.

For lower value $R{e}_{i}=110$ [see Fig. 9(II) (1a)–(2a)], a significant change of the torque profiles of helical states can be observed, specially at their minimum position. That is, the minimum values of $G(r)$ move towards the inner cylinder by increasing *Re*. As already seen for the profile of angular momentum, the torque curves of $mMC{S}_{{H}_{x}}$ mainly follow the trend of the wavy spiral solution. On the other hand, the torque profiles of the toroidally closed solutions become less pronounced by increasing their values at the center region. However, the profile for 8-$wTVF$ has significantly larger values in the plateau region. The torque profiles for helical states are very similar under both field configurations. The profiles of $L(r)$ of flow states with larger azimuthal wavenumber *m*, (for instance, *L*5-$wSP{I}_{{H}_{x}}$, *L*6-$wSP{I}_{{H}_{x}}$ and *L*7-$wSP{I}_{{H}_{x}}$ under a transversal field, and *L*5-*SPI* and *L*6-*SPI* under an axial field) are lifted up to the center region in the bulk [Fig. 9(I)]. At the same time, their profiles $G(r)$ show the maximum variation, *i*.*e*., the minimum to move towards the inner cylinder [Fig. 9(II)]. Physically, the axial mass flux is responsible for the minima of the curves $G(r)$ going towards the inner cylinder. However it is worth pointing out again that under a transverse magnetic field, the curves $G(r)$ presents the azimuthal averaged values [Fig. 9(II,2)]. Therefore, depending on the azimuthal position, the minima of $G(r)$ is shifted more or less towards the inner cylinder. Note that minimal radial distance is parallel to the applied transverse field and maximal distance is perpendicular to the field direction.

At larger values $R{e}_{i}=270$, the *parabola-like* shapes of the torque profiles $G(r)$ at the mid-gap region can be found and become more pronounced by increasing the dominant azimuthal wavenumber. When *Re* is increasing, parabola-like shapes of the torque profiles can be also found. Like the profiles of angular momentum, by increasing *Re*, the profiles of $G(r)$ show a monotonically varying trend with very little difference between these curves. The minimum of $G(r)$ of $wTV{F}_{l}$ and $wTV{F}_{l,{H}_{x}}$ move slightly towards outer region, and therefore the parabola shape of $G(r)$ becomes wider. But, for toroidal flows, the minimum of $G(r)$ move towards larger values of *r*.

The total torque *G*_total_ [insets in Fig. 9(II)] mainly enlarge within flow states incorporating larger azimuthal wavenumber *m* for toroidally closed or helical flow states, and also a similar phenomenon can be found when *Re* is increasing. That is, when flow structures itself become more energetic, *i*.*e*., for larger *Re* or larger internal velocities, the torque will be increased. An interesting finding is that there is a steep increase of *G*_*total*_ of $wTV{F}_{l}$ at $R{e}_{i}=270$, which may correspond to flow states having enlarged azimuthal wavenumber *m* [Fig. 9(II) (1b)]. At sufficient large *Re*, it even overcomes the corresponding value of *G*_total_ of the helical state. It means that the global transport becomes significantly enforced within the higher order *m* solutions under localized $wTV{F}_{l}$ structures.

### Miscellaneous structure

**Figure 10:**
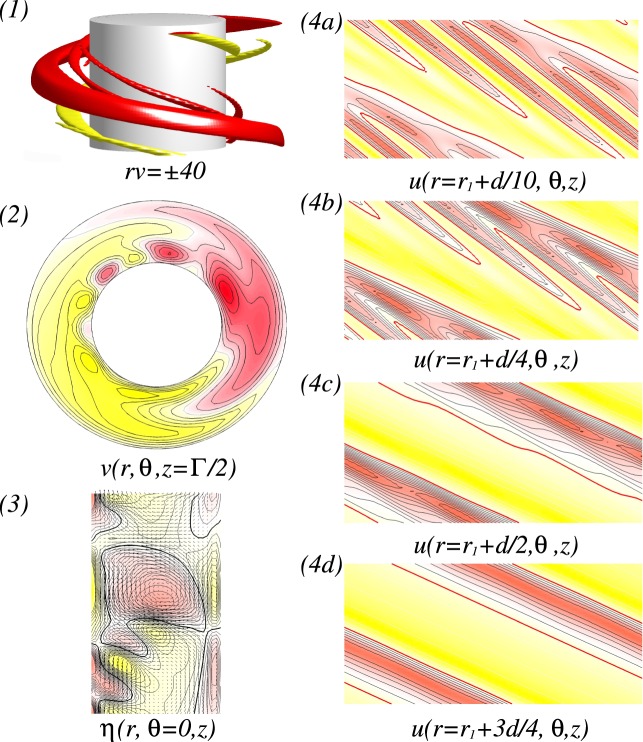
Flow visualizations of *L*1*L*2-*MCS*. Visualization of flow states *L*1*L*2-*MCS* as in Fig. 5, but for different parameters ${s}_{z}=0.6$, $R{e}_{i}=270$ and $Re=75$ (Fig. 10(1) clearly highlights both dominant azimuthal modes $m=1$ and $m=2$).

**Figure 11:**
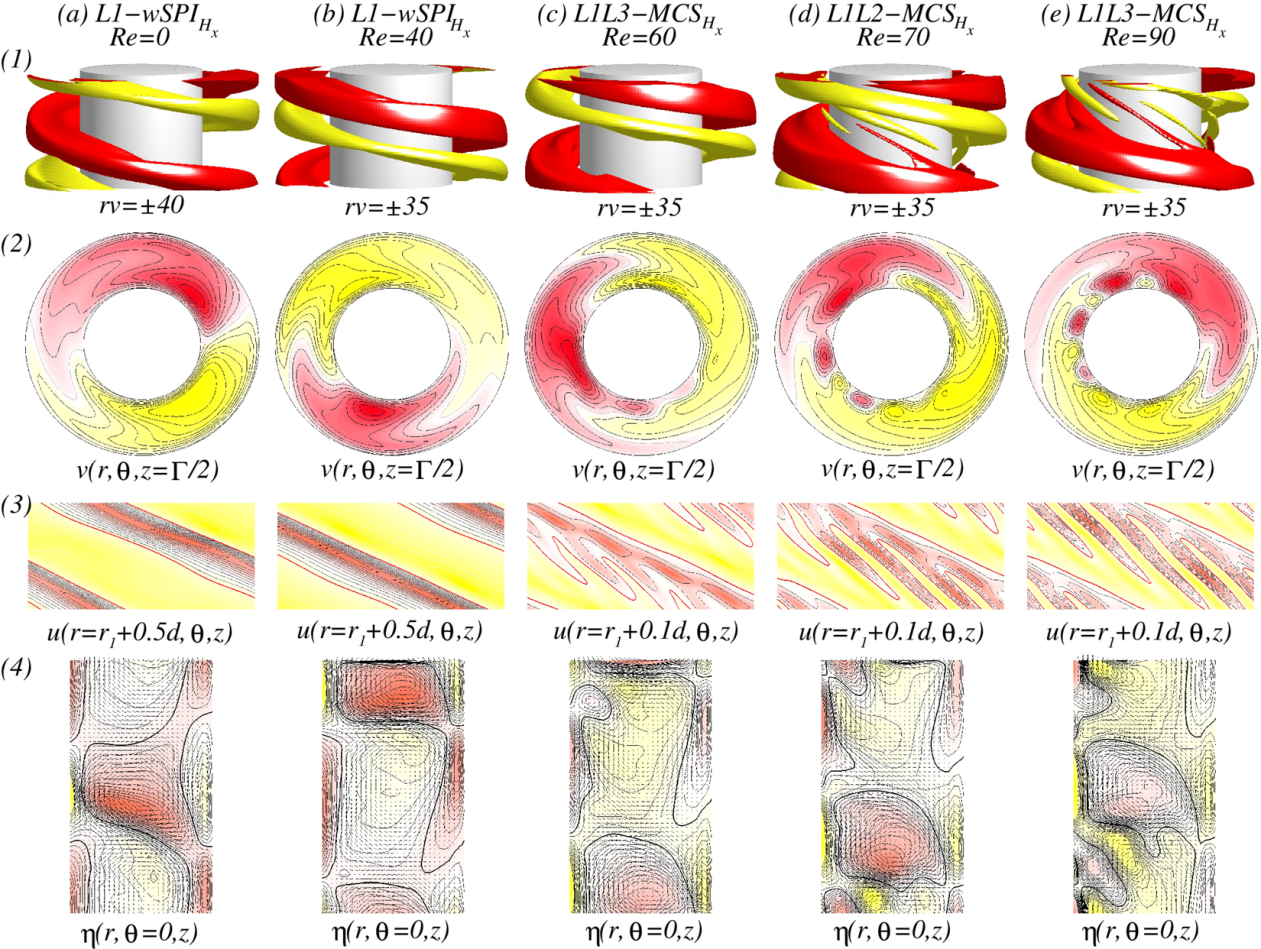
For $R{e}_{i}=270$, flow visualizations with *Re*. Flow visualization as in Fig. 8 but for helical flow states $wSP{I}_{{H}_{x}}$ and $MC{S}_{{H}_{x}}$ under a transverse field ${s}_{x}=0.6$. With increasing *Re* the second dominant azimuthal mode $m=2$ becomes more and more pronounced.

At $R{e}_{i}=270$, Fig. 10 shows a detailed flow structure of *L*1*L*2-*MCS*. That is, the flow pattern with larger azimuthal wavenumber $m=2$ (here not dominant) is located closer to the inner cylinder [see Fig. 10(4a) at $r=0.1d$], but the flow pattern with smaller dominant azimuthal wavenumber $m=1$ at the mid and outer region of the bulk [see Fig. 10(4c) and (4d)]. Figure 11 shows the analog flow patterns of *L*1*L*2-$MC{S}_{{H}_{x}}$ and *L*1*L*3-$MC{S}_{{H}_{x}}$, when a transverse field is applied. The $MC{S}_{{H}_{x}}$ occurred under a transverse magnetic field [Fig. 11(c–e)] is qualitatively identical to the *MCS* appeared under an axial magnetic field (Fig. 10) with a minor modulation due to the symmetry breaking effect of ${s}_{x}\ne 0$. Interestingly, for larger values *Re*, *L*1*L*2-$MC{S}_{{H}_{x}}$ first disappear against to *L*1*L*3-$MC{S}_{{H}_{x}}$, but finally returns back to *L*1*L*2-$MC{S}_{{H}_{x}}$. The flow pattern of $MC{S}_{{H}_{x}}$ corresponding to larger *m* is orientated close to the inner cylinder, but for smaller *m*, in the interior and outer bulk region [Fig. 11(c–e)]. From the vector plot $[u(r,z),w(r,z)]$ of $MC{S}_{{H}_{x}}$ (Figs 8 and 10), one can see an arrangement of vortices near the inner cylinder with an incline towards the bulk interior, which is similar to the scenario of the detected localized states *w*-$TV{F}_{l,{H}_{x}}$ [Fig. 8(4)].

The $MC{S}_{{H}_{x}}$ shown in Figs 8 and 10 present an interesting observation. As already mentioned, the larger azimuthal wavenumber is orientated closer towards the inner cylinder. Moreover, regarding the vector plot $[u(r,z),w(r,z)]$, one sees an arrangement of vortices near the inner cylinder with an incline towards the bulk interior, which can be similarly detected for the localized *w*-$TV{F}_{l,{H}_{x}}$ states [Fig. 8(4)].

**Figure 12:**
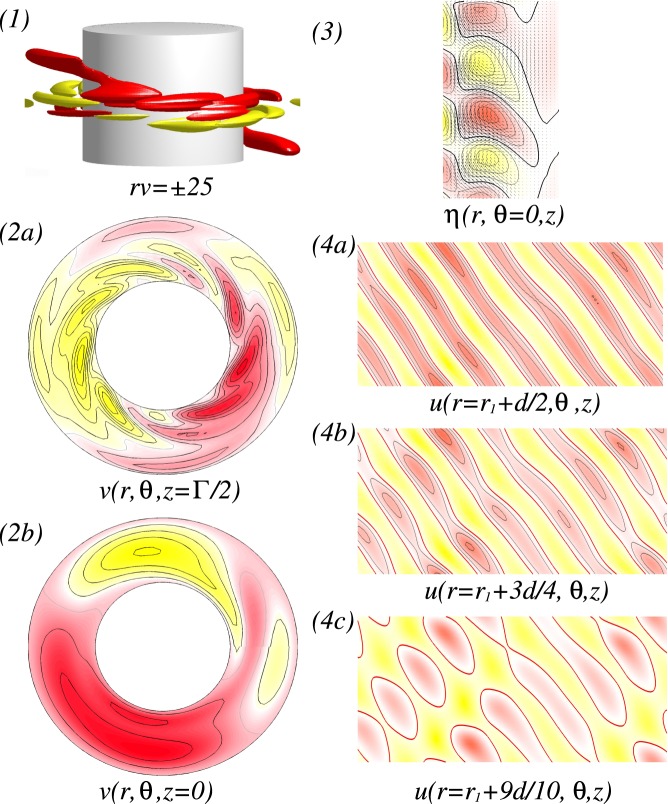
Flow visualizations of 8-1-$wTV{F}_{{H}_{x}}$. Flow visualization of flow states 8-1-$wTV{F}_{{H}_{x}}$ at ${s}_{x}=0.6$ & ${s}_{z}=0.0$, $R{e}_{i}=300$ in absence of axial through flow, *i*.*e*., $Re=0$. Shown are: (1) isosurface of *rv*; (2) the azimuthal velocity $v(r,\theta )$ within the 8-1-$wTV{F}_{{H}_{x}}$ structure (**a**) and half system length apart (**b**); (3) vector plot $[u(r,z),w(r,z)]$ of the radial (*u*) and axial (*u*) velocity components including color-coded azimuthal vorticity $\eta (r,\theta =0,z)$); (4a–c) contours of the radial velocity $u(r,\theta ,z)$ at different radial positions as indicated.

Under a transverse magnetic field, the 8-1-$wTV{F}_{{H}_{x}}$ state detected at large $R{e}_{i}$ (Fig. 12) looks similar to the localized 8-$wTV{F}_{l}$ (Fig. 8). But this flow has a strong addition modulation due to $m=1$ [Fig. 12(1)], which is clearly visible in the azimuthal velocity $v(r,\theta )$ within the wTVF structure and half system length apart [Fig. 12(2)], and in the radial velocity $u(r,\theta ,z)$ on an unrolled cylindrical surface [Fig. 12(4)]. Although the azimuthal wavenumber $m$ decreases from the inner to the outer bulk region, contours of the radial velocity [Fig. 12(4)] and vector plot $[u(r,z),w(r,z)]$ of the radial (*u*) and axial (*u*) velocity components clearly show that this flow structure does not have any kind of axial localization as $wTV{F}_{l}$ has.

## Discussion and Conclusion

As a foundational paradigm of fluid dynamics, the TCS has been extensively investigated computationally and experimentally for more than a century. In spite of the long history of the TCS and the vast literature on the topic, the dynamics of TCS with a complex fluid have begun to be investigated relatively recently. In this paper we investigate the effect of an externally imposed *axial mass flux* (*axial pressure gradient*, *axial through flow*) on ferrofluidic Taylor-Couette flow under the influence of magnetic fields. As far as we know, the study of effects of an axial mass flux on a ferrofluidic system is considered in this paper for the first time.

Through systematic and extensive simulations of the ferrohydrodynamical equations, a generalization of the classic Navier-Stokes equation into ferrofluidic systems subject to magnetic fields and an axial mass flux, we unveil the emergence and evolution of distinct and new flow states. That is, when an axial mass flux (axial through flow, described by Reynolds number *Re*) is applied to a ferrofludic system, the dynamics of a system can be described by results of competition of the *three* different instabilities; *centrifugal instability* due to rotation, *shear instability* due to axial mass flux and *magnetic instability* due to applied magnetic fields. Through a competition of these instabilities, *previously unknown new* flow states will be created or occurred, and also complicated dynamics with various flow structures can be produced. Finally, we found *new* flow states: *localized wavy Taylor vortices* ($wTV{F}_{l}$ and $wTV{F}_{l,{H}_{x}}$) and *modulated Mixed-Cross-Spirals* ($mMC{S}_{{H}_{x}}$). Note that the new found localized $wTV{F}_{l}$ and $wTV{F}_{l,{H}_{x}}$ differ from the classical ones due to change of azimuthal wavenumber with respect to the axial position. In general we find the azimuthal wavenumber of the localized structure always to be equal or larger than the one of the surrounding background flow. Note that as described in earlier studies^18,21,26,36^, in presence of a transverse magnetic field, all flow states are inherently three-dimensional, and *Mixed-Cross-Spirals* (Not modulated *MCS* and $MC{S}_{{H}_{x}}$, here with same chirality) was already found by one of the authors^47,48^.

The detailed emergence of various flow states and their transient behavior can be summarized as follows.

1. For low rotation value of the inner cylinder rotation, *Re*_*i*_ = 110:
  When an axial through flow *Re* is applied, *TVF* ($wTV{F}_{{H}_{x}}$) with the toroidally closed flow structure loses its stability, and then bifurcates to 1-$wTVF$ (2-$wTV{F}_{{H}_{x}}$) via a secondary supercritical Hopf bifurcation, respectively. For more strong axial through flow *Re*, 1-$wTVF$ (2-$wTV{F}_{{H}_{x}}$) becomes unstable, and then moves towards the only stable helical solution, *L*1-*SPI* (*L*1-$wSP{I}_{{H}_{x}}$), respectively. Under the influence of an axial or a transverse magnetic field, by increasing *Re*, this helical flow state will give raise to a bifurcation sequence whose azimuthal wavenumber *m* is continuously increasing from its helicity found in the absence of any magnetic field. Note that for the absolute/critical values of occurring different flow states, they are merely different, which is due to stabilizing effect of a magnetic field on the basic state. That is, for larger *Re*_*i*_, the shifting of the critical values will be produced. By decreasing *Re*, we observed *hysteretic* behavior at coexisting regions of two *SPI* [$SP{I}_{{H}_{x}}$] states. Note that for any magnetic field, we could not get any stable *L*2-*SPI* or *L*2-$wSP{I}_{{H}_{x}}$ state.
  Note that all flow states under the influence of a transverse field have additional finite $m\pm 2$ modes due to the symmetry breaking effect of its magnetic field. For the occurrence of the newly found state called the *stable modulated Mixed-Cross-Spirals* ($mMC{S}_{{H}_{x}}$), we found that these additional $m\pm 2$ modes is mainly contributed. Actually, the creation of new states is from an interaction between the $wSP{I}_{{H}_{x}}$ states and additional stimulated modes $m\pm 2$. For instance, for the fixed transverse field ${s}_{x}=0.6$, we only observe a stable $mMC{S}_{{H}_{x}}$ state between two $wSP{I}_{{H}_{x}}$ states with large azimuthal wavenumbers *m* (or strong helicity) for sufficient/fairly large value *Re*, but for smaller *Re*, we could not observe them as a stable state. In fact, we may temporarily detect $mMC{S}_{{H}_{x}}$ as *transient/interim* states between $wSP{I}_{{H}_{x}}$ with small *m* for small *Re*, which makes us speculate their existence as *unstable* states. Unfortunately it cannot be detected by our present numerical code. Besides this new flow state, we also detected the already known states, *Mixed-Cross-Spiral MCS* ($MC{S}_{{H}_{x}}$) with the same helicity of *SPI* ($wSP{I}_{{H}_{x}}$). Within this *MCS*s, the higher/larger azimuthal mode *m* can be always found to be closer oriented to the inner cylinder.
2. For high rotation value of the inner cylinder rotation, *Re*_*i*_ = 270:

In the presence of an axial magnetic field, we observed that as an effect of an axial through flow *Re*, a bifurcation sequence can be generated from the initial toroidally closed state $TVF$ to localized $wTVF$ ($TVF\to 3$-$wTV{F}_{l}\to 4$-$wTV{F}_{l}\to 5$-$wTV{F}_{l}\to 8$-$wTV{F}_{l}$), and their azimuthal wavenumber *m* is also increasing. We note that 2-$wTV{F}_{l}$, 6-$wTV{F}_{l}$ and 7-$wTV{F}_{l}$ can not be found as stable states, but detected as transient flow states, which is similar to the scenario of helical flow structures, discussed before. It means that these flow states can exist as *unstable* states.

When a transverse magnetic field is presented, we observed that an axial through flow affects $wTV{F}_{{H}_{x}}$ to evolve to 4-$wTV{F}_{l,{H}_{x}}$ with dominant azimuthal wavenumber $m=4$ and the background flows within $wTV{F}_{l}$ always have *equal* or *smaller* azimuthal wavenumber *m* compared to the localized structure. On the other hand, helical states/structures $(w)SPI{s}_{{H}_{x}}$ itself move towards the inner cylinder with increasing *m* and *Re*. The helical flow states existed in the absence of an axial through flow (${Re}=0$) are already wavy-modulated, but by increasing the axial through flow *Re*, the waviness can disappear. Finally, the state *L*1-*SPI* (*L*1-$SP{I}_{{H}_{x}}$) can be appeared for an axial (transverse) magnetic field, respectively. Depending on an axial through flow *Re*, we may detect various types of *MCS* with the growth of azimuthal modes *m*. For instance, see bifurcation sequence: $L1$-$wSP{I}_{{H}_{x}}\to L1L3$-$MC{S}_{{H}_{x}}\to L1L2$-$MC{S}_{{H}_{x}}\to L1L3$-$MC{S}_{{H}_{x}}$. Like classical cases of $MC{S}_{{H}_{x}}$, the new $mMC{S}_{{H}_{x}}$ state can bifurcate to stable or unstable solution which is connected to $wSP{I}_{{H}_{x}}$ as footbridge^48^ or bypass solutions^47^. Typically the $wTV{F}_{l,{H}_{x}}$ states only appear for sufficient large values *Re*, and also the exact values of such bifurcating points depend on a field strength or an orientation of the magnetic field.

In summary, we have shown the new flow state $mMC{S}_{{H}_{x}}$, called modulated Mixed-Cross-Spiral, as a byproduct of an interaction of an axial through flow and a transversal magnetic field. To show its dynamical properties, we consider quantities of flow states including a total kinetic energy, a dominant mode amplitude, flow visualization, a power spectral density, torque, angular momentum, etc. From the detection of the previously unknown new flow state $mMC{S}_{{H}_{x}}$, we also see that a symmetry breaking transverse magnetic field is responsible for the appearance of new flow structures due to its additionally stimulated modes $m\pm 2$. Our work allows some insights into the structure of complex stable flows having more or less strong variation of angular momentum and torque due to nonlinear interaction of magnetic particles and magnetic fields.

We hope that our computational results will stimulate experimental works on ferrofluidic flows under the influence of external applied mass flux, because the setting of our computation and the chosen parameters are in well accessible experimental regime. Specially, it may be feasible to realize the flow state $mMC{S}_{{H}_{x}}$ in experiments. Control of flow pattern through an axial through flow and the magnetic fields may be possible and also application to flow separation devices.

## Methods

### Ferrohydrodynamical equations of motion

As described in the manuscript, the non-dimensionalized hydrodynamical equations^21,37^ are given by:

9 $$\begin{array}{rcl}({\partial }_{t}+{\bf{u}}\cdot \nabla ){\bf{u}}-{\nabla }^{2}{\bf{u}}+\nabla p & = & ({\bf{M}}\cdot \nabla ){\bf{H}}+\frac{1}{2}\nabla \times ({\bf{M}}\times {\bf{H}}),\\ \nabla \cdot {\bf{u}} & = & 0.\end{array}$$

The boundary conditions on the cylindrical surfaces are given by ${\bf{u}}({r}_{i},\theta ,z)=(0,{R}{{e}}_{i},0)$ and ${\bf{u}}({r}_{o},\theta ,z)=(0,{R}{{e}}_{o},0)$, where the inner and the outer Reynolds numbers are $R{e}_{i}={\omega }_{i}{r}_{i}d/\nu$ and $R{e}_{o}=0$ (at rest), respectively. Here, ${r}_{i}={R}_{i}/({R}_{o}-{R}_{i})$ and ${r}_{o}={R}_{o}/({R}_{o}-{R}_{i})$ are the non-dimensionalized inner and outer cylinder radii, respectively.

We need to solve Eq. (9) together with an equation describing the magnetization of the ferrofluid. Using the equilibrium magnetization of an unperturbed state where homogeneously magnetized ferrofluid is at rest and the mean magnetic moment is orientated in the direction of the magnetic field, we have ${{\bf{M}}}^{{\rm{eq}}}=\chi {\bf{H}}$. The magnetic susceptibility $\chi$ of the ferrofluid can be approximated with the Langevin’s formula^50^, where we set the initial value of $\chi$ to be 0.9 and use a linear magnetization law. The ferrofluid studied corresponds to APG933^51^. We consider the near equilibrium approximations of Niklas^45,52^ with small $\parallel {\bf{M}}-{{\bf{M}}}^{{\rm{eq}}}\parallel$ and small magnetic relaxation time $\tau$: $|\nabla \times {\bf{u}}|\tau \ll 1$. Using these approximations, one can obtain^21^ the following magnetization equation:

10 $${\bf{M}}-{{\bf{M}}}^{{\rm{eq}}}={c}_{N}^{2}(\frac{1}{2}\nabla \times {\bf{u}}\times {\bf{H}}+{\lambda }_{2}{\mathbb{S}}{\bf{H}}),$$

where

11 $${c}_{N}^{2}=\tau /(1/\chi +\tau {\mu }_{0}{H}^{2}/6\mu \Phi )$$

is the Niklas coefficient^45^, *μ* is the dynamic viscosity, $\Phi$ is the volume fraction of the magnetic material, ${\mathbb{S}}$ is the symmetric component of the velocity gradient tensor^21,37^, and *λ*_2_ is the material-dependent transport coefficient^37^, which we choose to be ${\lambda }_{2}=4/5$^26,37,53^. Using Eq. (10), we can eliminate the magnetization from Eq. (9) to obtain the following ferrohydrodynamical equations of motion^21,37^:

12 $$({\partial }_{t}+{\bf{u}}\cdot \nabla ){\bf{u}}-{\nabla }^{2}{\bf{u}}+\nabla {p}_{M}=-\,\frac{{s}_{N}^{2}}{2}[{\bf{H}}\nabla \cdot ({\bf{F}}+\frac{4}{5}{\mathbb{S}}{\bf{H}})+{\bf{H}}\times \nabla \times ({\bf{F}}+\frac{4}{5}{\mathbb{S}}{\bf{H}})],$$

where ${\bf{F}}=(\nabla \times {\bf{u}}/2)\times {\bf{H}}$, *p*_*M*_ is the dynamic pressure incorporating all magnetic terms that can be expressed as gradients, and *s*_*N*_ is the Niklas parameter [Eq. (14)]. To the leading order, the internal magnetic field in the ferrofluid can be approximated as the externally imposed field^20^, which is reasonable for obtaining dynamical solutions of the magnetically driven fluid motion. Equation (12) can then be simplified as

13 $$\begin{array}{rcl}({\partial }_{t}+{\bf{u}}\cdot \nabla ){\bf{u}}-{\nabla }^{2}{\bf{u}}+\nabla {p}_{M} & = & {s}_{N}^{2}\{{\nabla }^{2}{\bf{u}}-\frac{4}{5}[\nabla \cdot ({\mathbb{S}}{\bf{H}})]\\  &  & -\,{\bf{H}}\times [\frac{1}{2}\nabla \times (\nabla \times {\bf{u}}\times {\bf{H}})-{\bf{H}}\times ({\nabla }^{2}{\bf{u}})\\  &  & +\,\frac{4}{5}\nabla \times ({\mathbb{S}}{\bf{H}})]\}.\end{array}$$

This way, the effect of the magnetic field and the magnetic properties of the ferrofluid on the velocity field can be characterized by a single parameter, the magnetic field or the Niklas parameter^45^, ${s}_{N}^{2}={s}_{x}^{2}$ $[{s}_{z}^{2}]$, with

14 $${s}_{x}=\frac{2(2+\chi ){H}_{x}{c}_{N}}{{(2+\chi )}^{2}-{\chi }^{2}{\eta }^{2}},\,{\rm{and}}\,{s}_{z}={H}_{z}{c}_{N},$$

for transversal and axial magnetic field, respectively.

## Supplementary information

Movie 1

Movie 2

Movie 3

Supplementary material

## Footnotes

1 ^1^Basic state is a 2-fold modified CCF due *m* = ±2 stimulation in transverse field. Thus we prefer 2-AVF as the flow is inherent 3D in contract to classical 2D CCF.
