# Supplementary material for: Effects of an imposed axial flow on a Ferrofluidic Taylor-Couette flow
Source: Sci Rep. 2019 Oct 28;9:15438. doi: 10.1038/s41598-019-51935-x (PMC6817883; doi:10.1038/s41598-019-51935-x)
Supplement: Supplementary file 4 — Supplementary material [file 41598_2019_51935_MOESM4_ESM.pdf]

Supplementary Material to
"Effects of an imposed axial flow on a Ferrofluidic Taylor-Couette flow"

Sebastian Altmeyer[1] and Younghae Do[2,*]

[1]Castelldefels School of Telecom and Aerospace Engineering, Universitat Politècnica de Catalunya,08034 Barcelona, Spain

[2]Department of Mathematics, KNU-Center for Nonlinear Dynamics, Kyungpook National University, Daegu, 41566, Republic of Korea

(Dated: July 4, 2019)

Legends for videos in SM

- MovieA1:
  MovieA1 demonstrates the spatio-temporal evolution of L6L5L4-MCS$_{H_x}$ at $Re_i = 110$, $Re_o = 0$, $Re = 82$ and $s_x = 0.6$. *Top left:* Isosurfaces of the angular momentum $rv = \pm 15$ [red (yellow) color indicates positive (negative) angular momentum]. *Bottom left:* Contours of azimuthal velocity component v in the (r, θ) plane at mid-height (viewed from the bottom). *Top middle:* Vector plots $[u(r, z), w(r, z)]$ of the radial and axial velocity components (including azimuthal vorticity $\eta(r, \theta)$) at $\theta = 0$. Red (yellow) [dark (light gray)] contours correspond to positive [(negative)] values. Radial velocity $u(\theta, z)$ on an unrolled cylindrical surface in the annulus at (*Top right:*) mid-gap ($r = d/2$) and (*Bottom right:*) a quarter gap width away from the outer cylinder ($r = 3d/4$) [red (yellow) color indicates in (out) flow]. Thick black arrows are highlighting the rotation direction of the inner cylinder.

- MovieA2:
  MovieA2 demonstrates the spatio-temporal evolution of 5-wTVF$_l$ at $Re_i = 270$, $Re_o = 0$, $Re = 55$ and $s_z = 0.6$. *Top left:* Isosurfaces of the angular momentum $rv = \pm 15$ [red (yellow) color indicates positive (negative) angular momentum]. *Bottom left:* Contours of azimuthal velocity component v in the (r, θ) plane at mid-height (viewed from the bottom). *Top middle:* Vector plots $[u(r, z), w(r, z)]$ of the radial and axial velocity components (including azimuthal vorticity $\eta(r, \theta)$) at $\theta = 0$. Red (yellow) [dark (light gray)] contours correspond to positive [(negative)] values. *Top right:* Radial velocity $u(\theta, z)$ on an unrolled cylindrical surface in the annulus at mid-gap ($r = d/2$) [red (yellow) color indicates in (out) flow]. Thick black arrows are highlighting the rotation direction of the inner cylinder.

- MovieA3:
  MovieA3 demonstrates the spatio-temporal evolution of 2-wTVF$_{l,H_x}$ at $Re_i = 270$, $Re_o = 0$, $Re = 50$ and $s_x = 0.6$. *Top left:* Isosurfaces of the angular momentum $rv = \pm 15$ [red (yellow) color indicates positive (negative) angular momentum]. *Bottom left:* Contours of azimuthal velocity component v in the (r, θ) plane at mid-height (viewed from the bottom). *Top middle:* Vector plots $[u(r, z), w(r, z)]$ of the radial and axial velocity components (including azimuthal vorticity $\eta(r, \theta)$) at $\theta = 0$. Red (yellow) [dark (light gray)] contours correspond to positive [(negative)] values. *Top right:* Radial velocity $u(\theta, z)$ on an unrolled cylindrical surface in the annulus at mid-gap ($r = d/2$) [red (yellow) color indicates in (out) flow]. Thick black arrows are highlighting the rotation direction of the inner cylinder.

* Electronic address: yhdo@knu.ac.kr
